# Evaluation of cell-cell interaction methods by integrating single-cell RNA sequencing data with spatial information

**DOI:** 10.1186/s13059-022-02783-y

**Published:** 2022-10-17

**Authors:** Zhaoyang Liu, Dongqing Sun, Chenfei Wang

**Affiliations:** 1 Key Laboratory of Spine and Spinal Cord Injury Repair and Regeneration of Ministry of Education, Department of Orthopedics, Tongji Hospital, School of Life Science and Technology, Tongji University, Shanghai, 200092 China; 2 Frontier Science Center for Stem Cells, School of Life Sciences and Technology, Tongji University, Shanghai, 200092 China

**Keywords:** Cell-cell interaction, Benchmarking, scRNA-seq, Spatial transcriptomics, Spatial interaction tendency

## Abstract

**Background:**

Cell-cell interactions are important for information exchange between different cells, which are the fundamental basis of many biological processes. Recent advances in single-cell RNA sequencing (scRNA-seq) enable the characterization of cell-cell interactions using computational methods. However, it is hard to evaluate these methods since no ground truth is provided. Spatial transcriptomics (ST) data profiles the relative position of different cells. We propose that the spatial distance suggests the interaction tendency of different cell types, thus could be used for evaluating cell-cell interaction tools.

**Results:**

We benchmark 16 cell-cell interaction methods by integrating scRNA-seq with ST data. We characterize cell-cell interactions into short-range and long-range interactions using spatial distance distributions between ligands and receptors. Based on this classification, we define the distance enrichment score and apply an evaluation workflow to 16 cell-cell interaction tools using 15 simulated and 5 real scRNA-seq and ST datasets. We also compare the consistency of the results from single tools with the commonly identified interactions. Our results suggest that the interactions predicted by different tools are highly dynamic, and the statistical-based methods show overall better performance than network-based methods and ST-based methods.

**Conclusions:**

Our study presents a comprehensive evaluation of cell-cell interaction tools for scRNA-seq. CellChat, CellPhoneDB, NicheNet, and ICELLNET show overall better performance than other tools in terms of consistency with spatial tendency and software scalability. We recommend using results from at least two methods to ensure the accuracy of identified interactions. We have packaged the benchmark workflow with detailed documentation at GitHub (https://github.com/wanglabtongji/CCI).

**Supplementary Information:**

The online version contains supplementary material available at 10.1186/s13059-022-02783-y.

## Background

Cell-cell interactions (CCIs) are essential to various biological processes in multicellular organisms [1]. The biological behavior of a cell is regulated by its intracellular regulatory network and extracellular signaling environment at the same time and can eventually decide the function of that cell [2, 3]. In the multicellular interacting network, cells can interact and influence each other’s behavior through specific signaling molecules, including ligands, receptors, metabolites, ions, and structural or secreted proteins [4], leading to dynamic changes in cellular functions. Understanding how the cells interact with each other will help to reveal the potential mechanisms behind biological processes, such as organ development [5, 6] and tumor progression [7, 8].

With the advanced development of single-cell RNA sequencing (scRNA-seq), several computational tools are designed to infer CCIs through integrating gene expression from scRNA-seq data and ligand-receptor information [3, 4, 9]. These CCI tools rely on different models to simulate the background and evaluate the enrichment of ligand-receptor interactions over the background, which could be largely classified into 3 types (Table 1). The first type of method, statistical-based CCI tools, applies a statistical test to quantify the probability of each interaction over null hypotheses, such as CellPhoneDB [10] and CellChat [11]. The second type, network-based CCI tools, uses a more complex network model to weigh ligand-receptor interactions between cell types. For example, NicheNet integrates the intracellular gene regulatory information into the network model for better evaluating the possibilities of CCIs [12]. The third type, ST-based CCI tools, integrate spatial information to correct interactions predicted by gene expression, for instance, CellPhoneDB v3 [13] only focuses on interactions between cell types in the same spatial microenvironment. Although these CCI tools can infer a series of interactions using the annotated scRNA-seq data, a key question remains that the accuracy of inferred CCIs is not tested since there is no golden standard dataset for benchmarking them [4, 14]. Experimental validate these interactions is labor-intensive and only a few pairs can be validated at the same time. Alternative strategies that could benchmark the accuracy of predicted CCIs are in great demand.

**Table 1:** CCI tools included in this study

Tools	Method	Subunit	Prior knowledge	Language	Ref.
**Statistical-based tools**					
CellCall	Embedded pathway activity analysis for activity score; hypergeometric testing for significance of pathway activity	Single subunit	Ligand-receptor pairs; downstream TF regulation	R	[15]
CellChat	Law of mass action for communication probability; permutation test for significant interactions	Multi-subunit	Ligand-receptor pairs; signaling cofactors and pathways	R	[11]
CellPhoneDB	The mean of average ligand and receptor expression values for interaction enrichment; permutation test for significant interactions	Multi-subunit	Ligand-receptor pairs	Python	[10]
ICELLNET	Product of ligand and receptor expression values for communication score; geometric mean for multi-subunit complexes; Wilcoxon statistical test for highly potential interactions	Multi-subunit	Ligand-receptor pairs	R	[16]
iTALK	Finding differentially expressed ligand and receptor genes between cell types	Single subunit	Ligand-receptor pairs	R	[17]
SingleCellSignalR	Regularized product of ligand and receptor for lr-score; estimate lr-score cutoff for filtering interactions	Single subunit	Ligand-receptor pairs	R	[18]
**Network-based tools**					
Connectome	Cell types as nodes, interactions as edges; gene-wise *z*-score of ligand and receptor expression values as edge weights; system-wide Wilcoxon rank sum test for significant edges filtering	Single subunit	Ligand-receptor pairs	R	[19]
CytoTalk	Integrate two de novo intracellular signaling networks by known ligand-receptor interactions; optimal subnetwork searching for significant interactions	Single subunit	Ligand-receptor pairs	R	[20]
Domino	Construction global signaling network; cluster specific signaling subnetwork for prediction	Multi-subunit	Ligand-receptor pairs; TF regulation	R	[21]
NATMI	Cell types as nodes, interactions as edges; mean expression or specificity for edge weights; edge weight ranks for confident interactions	Single subunit	Ligand-receptor pairs	Python	[22]
NicheNet	Weighted network prior knowledge model; compute ligand activity and regulatory potential score using network propagation; select interactions by potential score	Single subunit	Ligand-receptor pairs; ligand-target pairs; receptor-target pairs	R	[12]
scMLnet	Construct primary ligand-receptor, TF-target, receptor-TF subnetworks using highly expressed genes; merge three subnetworks as final output	Single subunit	Ligand-receptor pairs; receptor-TF pairs; TF-target pairs	R	[23]
**ST-based tools**					
CellPhoneDB v3	L-R expression for enrichment; permutation test for significance; filter interactions based on spatial microenvironment	Multi-subunit	Ligand-receptor pairs; spatial microenvironment	Python	[13]
Giotto	Spatial proximity for interacting cell types; spatial co-expression for interactions	Single subunit	Ligand-receptor pairs; cell type colocalization; L-R co-expression	R	[24]
stLearn	Identify interactions by L-R co-expression and cell type density	Single subunit	Ligand-receptor pairs; cell type colocalization; L-R co-expression	Python	[25]

It is well-known that CCIs in the tissue environment are strongly determined by the spatial structures [26], and knowing the spatial positions between different cells can provide further information for their interaction possibilities and is independent of gene expression. In general, CCIs can be classified into 4 types, autocrine, juxtacrine, paracrine, and endocrine with increasing signaling ranges [4]. Among them, autocrine and juxtacrine only occur on cells themselves or contacted cells, while paracrine and endocrine could have spatially long-range effects. The recent development of spatial transcriptomics (ST) enables the recording of gene expression and spatial position of cells at the same time [26, 27], which could be used to characterize the spatial distance tendency of known interactions. While most CCI tools predict the interactions only using scRNA-seq, the consistency of interaction’s spatial distance tendencies and the actual spatial cell type distribution could be used to evaluate the possibility of inferred CCIs. For example, juxtacrine relies on the physical contact between cells, indicating that juxtacrine-type CCI has a high tendency to happen in spatially adjacent cells [4]. If a CCI tool predicted such juxtacrine-type CCIs between two spatially distal cell types, the predicted CCIs will have low confidence since juxtacrine-type CCIs cannot occur on noncontact cells.

Here, we defined and validated the spatial distance tendencies of known ligand-receptor interactions using ST data and separated them into long-range and short-range interactions. Based on this, we developed a method to evaluate CCI tools using the coherence between expected and observed spatial distance tendencies by integrating scRNA-seq and ST data. We also evaluated the consistency of predicted CCIs between different methods. We applied the benchmark method on 16 CCI tools in 15 simulated and 5 real datasets and summarizes these results to rank their performances. Finally, we evaluated the running time and memory efficiency of different tools. Our results suggest that the statistical-based method show in general better performance, and integrating results from two or more CCI tools will generate high-confidence CCI predictions.

## Results

### Definition of interactions ranges for ligand-receptor pairs by spatial transcriptomics data

**Fig. 1:**
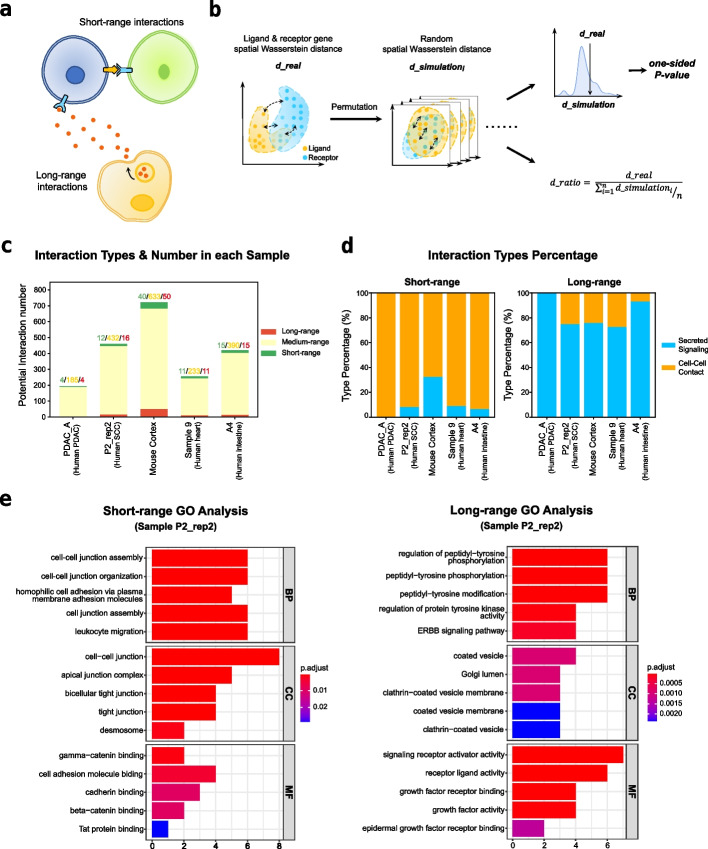
Defining and validating short-range and long-range interactions. **a** The illustration of short-range and long-range interactions. **b** The workflow of defining short-range and long-range interactions. First, generate spatial ligand and receptor gene distributions from ST data and calculate the Wasserstein distance between them. Next, perform permutation test on the Wasserstein distance to get the interaction’s spatial tendency and its confidence for filtering short-range and long-range interactions. *d_real*: the actual Wasserstein distance between ligand and receptor gene distributions; *d_simulation*: the Wasserstein distance between two permuted ligand and receptor gene distributions; *d_ratio*: the ratio of actual Wasserstein distance and average permuted Wasserstein distance, indicating the spatial tendency of interaction; one-sided *P*-value: indicate the confidence of interaction’s spatial tendency. **c** The actual numbers of short-range, medium-range, and long-range interactions in each sample. Color for interaction tendency type, green: short-range; yellow: medium; red: long-range. PDAC: pancreatic ductal adenocarcinoma; SCC: squamous cell carcinoma. **d** The interaction type proportion in short-range and long-range interactions. Color for interaction type, blue: secreted signaling type interaction; orange: cell-cell contact type interaction. **e** The GO analysis results of short-range (left) and long-range (right) interactions’ ligands in the sample P2_rep2 in the SCC dataset

To define the interaction range, we first extracted all the ligand-receptor interactions in CellChatDB [11], a ligand-receptor database used in CellChat. Interactions with multi-subunit complexes were rearranged into single-subunit interactions (see “Methods”). CCIs can be separated into 4 types based on their interaction distance including autocrine, juxtacrine, paracrine, and endocrine [4]. However, it is hard to evaluate endocrine in the local tissue environment, and the autocrine also cannot be easily separated from juxtacrine since both of them have a relatively short interaction range. Considering this, we just separate all the ligand-receptor interaction pairs into short-range and long-range interactions instead of 4 types (Fig. 1a, see “Methods”).

We collected 5 different ST datasets to define the range of the interactions comprehensively, including a human pancreatic ductal adenocarcinoma (PDAC) dataset [28] and a human squamous cell carcinoma (SCC) dataset [29] from the tumor microenvironment (TME), a mouse cortex dataset [30] from the nervous system and a human heart dataset [31], and a human intestinal dataset [32] to represent the developmental system setting, all of these data were generated with matched scRNA-seq. For each sample in the ST dataset, we applied a permutation-based procedure to identify sample-specific short-range and long-range interactions (Fig. 1b). The spatial distribution distance of each ligand and receptor gene could be measured by the Wasserstein distance, a metric commonly used to define the distance between probability distributions. Then we permutated the spot position on the ST slides randomly and calculated the ratio of the real Wasserstein distance and permutated distance between ligand and receptor distributions as *d* _ *ratio*. Finally, we generated a distribution of *d* _ *ratio* using all ligand-receptor pairs and filtered the reliable short-range and long-range interactions based on the distribution and significance of the *P*-value (Fig. 1b, see “Methods”).

We next compared the identified short-range and long-range interactions between different samples. The actual number of filtered short-range and long-range interactions varied across samples (Fig. 1c) and is positively correlated with the gene coverage of the sample (Additional file 1: Fig. S1a-b). Although we could observe short-range and long-range interactions in each sample, the number is relatively small compared to the number of all interactions (Fig. 1c and Additional file 1: Fig. S1c). This result suggested that the spatial distance tendency of CCIs consistently existed among different biological systems, but only a few ligand-receptor pairs represented a significant and constant tendency among different cell types.

### Short-range and long-range interactions have distinct biological features

We next validated the functional features of the identified short-range and long-range interactions. Interactions in CellChatDB are classified into cell-cell contact and secreted signaling based on their known protein structures and biological pathways. We compared the enrichment of short-range and long-range interactions in different ligand-receptor categories. Not surprisingly, more than 90% percentage of the short-range interactions belong to the cell-cell contact type except for the mouse cortex, while long-range interactions are mostly secreted signaling (Fig. 1d). We further performed gene ontology (GO) enrichment analysis on the ligand genes of short-range and long-range interactions. In the sample P2_rep2 from the SCC dataset, the short-range interaction genes were enriched in cell-cell junction-associated biological processes and cellular components, including cell-cell junction assembly and cell adhesion molecule binding, which play a crucial role in regulating cell contact formation and stability [33, 34] (Fig. 1e). By contrast, long-range interaction genes were enriched in many signaling pathways that have a wide regulatory range, such as the ERBB signaling pathway [35] (Fig. 1e). Similar functional enrichments for short-range and long-range interactions are observed in other datasets. Taken together, these analyses suggest that the classification of short-range and long-range interactions could accurately reflect their interaction distance and functional properties, which could serve as a basis for our followed evaluations.

### CCI evaluation workflow

**Fig. 2:**
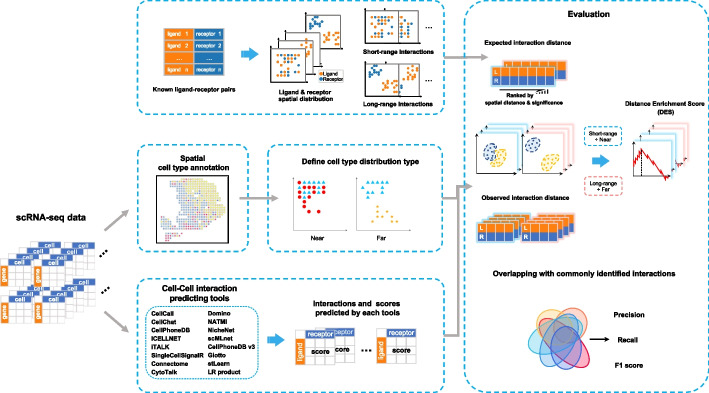
Schematic overview of evaluation workflow. First, generate known ligand-receptor pairs from CellChatDB, then select short-range and long-range interactions from known pairs for each dataset (top left). Next, perform spatial cell type annotation on ST data coupled with matched scRNA-seq data, and define near and far distributed cell type pairs based on the annotation (medium left). Then feed annotated scRNA-seq data to CCI tools and extract predicted results (bottom left). Finally, evaluate tools’ performances on both distance enrichment score and the metric of commonly identified interactions

We designed a comprehensive workflow to evaluate the accuracy of inferred CCIs from scRNA-seq data based on the expected and observed spatial distance tendencies (Fig. 2 and Additional file 1: Fig. S2). The short-range and long-range interactions defined using ST data could serve as expected spatial distance tendencies. Next, we applied 15 widely used CCI tools and a baseline method based on the product of L-R expression (named LR product) to infer CCIs. These 15 CCI tools include statistical-based methods such as CellCall [15], CellChat [11], CellPhoneDB [10], ICELLNET [16], iTALK [17], and SingleCellSignalR [18]; network-based methods such as Connectome [19], CytoTalk [20], Domino [21], NATMI [22], NicheNet [12], and scMLnet [23]; and ST-based methods such as CellPhoneDB v3 [13], Giotto [24], and stLearn [25] (Table 1). As scRNA-seq does not have the spatial information, we integrated them with matched ST dataset and evaluated the cell type distribution distance using the relative positions of spots from ST, which could serve as the observed spatial distance tendencies (Fig. 2). The consistency between expected and observed spatial tendencies could be used to evaluate the possibility of CCIs to happen in the real tissue environment, for which were calculated using a distance enrichment score (DES) similar to the ES score used in gene set enrichment analyses [36] (see “Methods”). Besides the consistency with spatial distance tendencies, we also defined the overlapped interactions shared by multiple CCI tools as a standard to evaluate the similarity of results from different tools (Fig. 2, see “Methods”). Finally, we summarized the DES metrics and F1 score to common interactions to evaluate the performance of CCI tools.

### Evaluation using simulated datasets

**Fig. 3:**
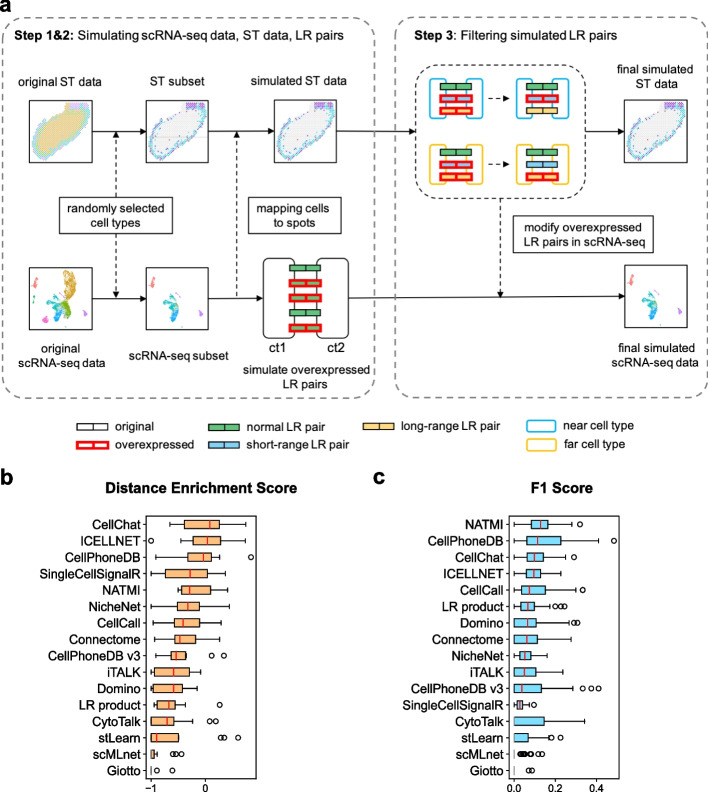
Evaluation using simulated datasets. **a** The schematic illustration of data simulation procedure. The long box indicates the LR pair between two cell types, where its face color represents interaction type and edge color represents expression state. The rounded rectangles indicate cell types and edge color represents distance type between cell types. **b** The DES ranks of CCI tools evaluating using 15 simulated datasets. Tools are sorted by the median DES. **c** The F1 ranks of CCI tools evaluating using 15 simulated datasets. Using simulated interactions as the positive set. Tools are sorted by the median F1 score

To demonstrate our DES metric could accurately reflect the performance of CCI tools, we simulated 15 paired scRNA-seq and ST data with overexpression of known ligand-receptor pairs. In each round of the simulation, we randomly selected four cell types and subseted original scRNA-seq and ST data based on selected cell types. Then we randomly mapped cells to ST spots according to their cell types and replace the original expressions in ST data. Next, L-R pairs will be randomly selected for each cell type pair and their original expression signals will be replaced with overexpressed signals using a semi-synthetic method [37]. Finally, we filtered simulated CCIs to keep short/long-range interaction consistent with the spatial distance of cell type pairs and generated the final simulated scRNA-seq and ST data (Fig. 3a, Additional file 1: Fig. S3, see “Methods”). For the 3 different biological systems used in our study (TME, nervous and developmental system), we selected one sample of data for each biology system and performed 5 round simulations for each sample. Finally, we evaluated these tools using 15 simulated datasets (Fig. 3b, c). CellChat, ICELLNET, and CellPhoneDB displayed high-ranked DES, while Giotto, scMLnet, and stLearn showed relatively poor performance (Fig. 3b). We also evaluated these tools by simply comparing the overlaps between predicted CCIs with simulated CCIs. Using simulated CCIs in each cell type pair as the positive set, we computed the F1 score for each tool. Consistently, CellPhoneDB, CellChat, and ICELLNET still show high F1 score ranks similar to DES rank (Fig. 3c). Interestingly, the statistical-based tools tend to have better performance than network-based or ST-based tools on simulated datasets. Taken together, these results suggest that our DES metric is comparable to the F1 score of ground truth in measuring the performance of different CCI tools.

### Evaluation using real datasets

#### Human pancreatic ductal adenocarcinomas (PDAC) dataset

**Fig. 4:**
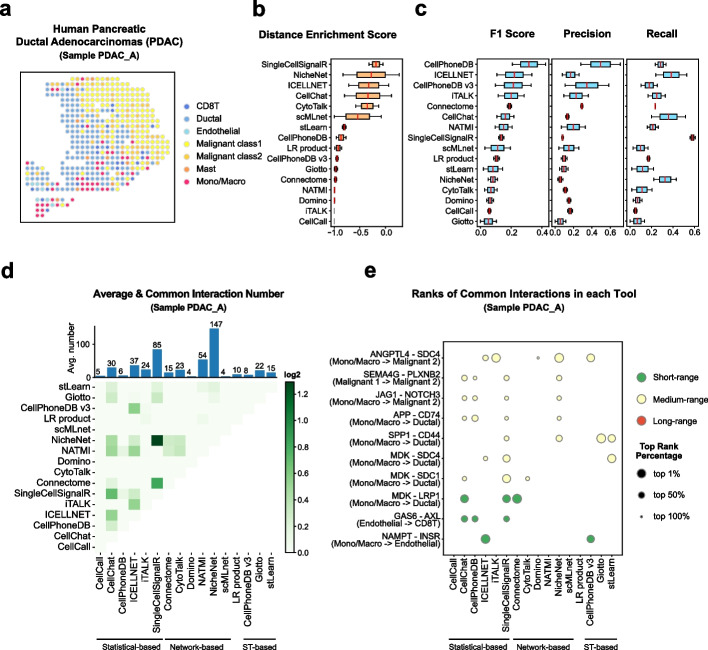
Evaluation results in the human PDAC dataset. **a** Spatial cell type annotation result of ST data of sample PDAC_A in the human PDCA dataset. Color indicates the spot’s cell type. **b** The box plot of tools’ average distance enrichment scores (DES) evaluated using all samples (PDAC_A, PDAC_B) in the human PDAC dataset, sorted by the median DES. **c** The box plots of tools’ average relative accuracy metrics (F1 score, precision, recall) evaluated using all samples (PDAC_A, PDAC_B) in the human PDAC dataset, all sorted by the median F1 score. **d** The heatmap shows the average common interaction number between each tool’s result per cell type in the sample PDAC_A. Color displays the interaction number. The bar plot on the top shows the average interaction number predicted by each tool per cell type pair. The actual number is labeled on the top of each bar. **e** The dot plot of top 10 most common interactions among all tools in the sample PDAC_A, sorted by interaction’s common count. The dot size indicates the top rank percentage of the interaction in corresponding tool’s result. The dot color reflects interaction’s spatial distance tendency in the sample PDAC_A. The CCI tools are grouped by their model types

The first dataset we used is from the PDAC tumor microenvironment. Using the matched scRNA-seq data with cell type annotations (Additional file 1: Fig. S4a), we annotated the spots in ST data of sample PDAC-A into 7 cell types (Fig. 4a and Additional file 1: Fig. S4b), including CD8^+^ T cells, ductal cells, endothelial cells, two cancer cell populations (Malignant class1, Malignant class2), mast cells, and monocytes or macrophages.

We detected a total of 193 ligand-receptor interactions in the PDAC-A sample (Fig. 1c). Among these interactions, there were 7 short-range interactions and 7 long-range interactions used for evaluation (Additional file 1: Fig. S1c and Fig. S4c). Reassuringly, the distributions of ligand and receptor expression had large overlapped areas for short-range interactions such as EFNB1–EPHB4, while for long-range interactions such as POSTN– (ITGAV+ITGB5), the ligand and receptors were distributed away from each other (Additional file 1: Fig. S4d). We next defined 11 near cell type pairs and 4 far cell type pairs by integrating scRNA-seq with ST data, which could be used as the distance for inferred CCIs (Additional file 1: Fig. S4e-f). The DES could be calculated as the enrichment of distance for inferred CCIs over the expected interaction ranges (see “Methods”). SingleCellSignalR, NicheNet, ICELLNET, CellChat, and CytoTalk showed a high-ranked DES, while CellCall, iTALK, and Domino are not consistent with spatial information (Fig. 4b, averaged result using all 2 PDAC samples). For similarity metrics with commonly identified interactions, CellPhoneDB, ICELLNET, and CellPhoneDB v3 were top-ranked by F1 score, and Giotto had the lowest F1 score (Fig. 4c). CellPhoneDB and CellPhoneDB v3 ranked first and second in precision across all the other tools, indicating high specificities of their results. As for the recall, SingleCellSignalR and ICELLNET performed well, which meant that they have a higher ability in identifying common interactions in the PDAC dataset. We also noticed that most of the tools with the top-ranked F1 score were statistical-based tools while network-based and ST-based tools had a generally lower F1 score. Several network-based tools such as NicheNet and CytoTalk showed a high-ranked DES but they failed to find more commonly identified interactions. This result suggested that the commonly identified interactions were likely biased dependent on the selected methods.

We visualized the overall predicted interaction networks of all cell types using the numbers of interactions, and most of the CCI tools shared a similar pattern (Additional file 1: Fig. S4g). For example, the Mono/Macro and ductal cells were commonly thought to be the major interacting cell types since the segments of these two cell types had the biggest sizes across most tools (Additional file 1: Fig. S4g, pink and sky blue). The patterns of Connectome, scMLnet, and Giotto were quite different from other tools and failed to find interactions between several cell types. For example, the interactions of malignant class 2 cells as receivers were totally missed in the scMLnet result. These analyses may also explain why scMLnet and Connectome did not get a high rank in relative accuracy metrics. Although most CCI tools shared an overall similar interaction pattern, the exact number of predicted interactions and common interactions varied largely across tools (Fig. 4d). NicheNet predicted the largest number of interactions per cell type (147), while Domino and scMLnet only have very few predicted CCIs even we tuned the parameters (4) (Fig. 4d).

We next focused on specific interactions and analyzed the top 10 common interactions among different tools (Fig. 4e). The most common interaction was ANGPTL4–SDC4, which interacted from Mono/Macro cells to malignant class2 cells; 5 tools could successfully predict it. Besides that, only 5 interactions existed in more than 3 tools’ results. This again reflected that the results between different tools varied largely. In addition, the statistical-based tools contributed to common interactions more than network-based tools. CellChat, CellPhoneDB, and SingleCellSignalR had similar patterns of identified interactions. Meanwhile, CellCall, Domino, NATMI, and scMLnet had no overlap between top common interactions. Interestingly, the top 10 common interactions that we found in the sample PDAC_A are all short-range and medium-range interactions, with no long-range interactions, indicating a relative cold TME (Fig. 4e). Our analyses showed that the statistical-based methods have an overall better performance in the PDAC TME data.

#### Human squamous cell carcinoma (SCC) dataset

**Fig. 5:**
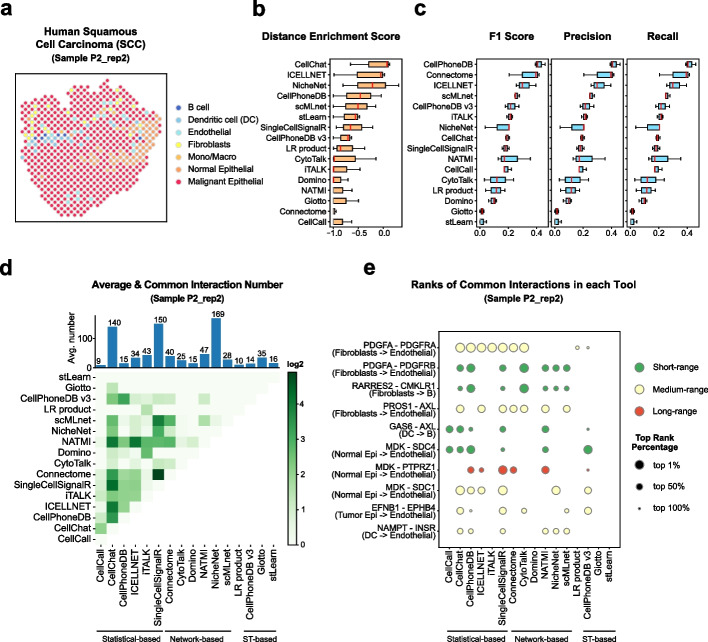
Evaluation results in the human SCC dataset. **a** Spatial cell type annotation result of ST data of sample P2_rep2 in the human SCC dataset. Color indicates the spot’s cell type. **b** The box plot of tools’ average distance enrichment scores (DES) evaluated using all samples (P2_rep2, P5_rep3, P10_rep1) in the human SCC dataset, sorted by the median DES. **c** The box plots of tools’ average relative accuracy metrics (F1 score, precision, recall) evaluated using all samples (P2_rep2, P5_rep3, P10_rep1) in the human SCC dataset, all sorted by the median F1 score. **d** The heatmap shows the average common interaction number between each tool’s result per cell type in the sample P2_rep2. Color displays the interaction number. The bar plot on the top shows the average interaction number predicted by each tool per cell type pair. The actual number is labeled on the top of each bar. **e** The dot plot of top 10 most common interactions among all tools in the sample P2_rep2, sorted by interaction’s common count. The dot size indicates the top rank percentage of the interaction in corresponding tool’s result. The dot color reflects interaction’s spatial distance tendency in the sample P2_rep2. The CCI tools are grouped by their model types

Next, we applied our benchmark workflow on another TME dataset from the SCC, here using sample P2_rep2 for demonstration. We identified 7 cell types in ST data using its paired scRNA-seq data, including B cell, dendritic cell, endothelial, fibroblasts, a merged cluster for monocytes and macrophages, and two types of epithelial (normal and malignant epithelial, Fig. 5a, Additional file 1: Fig. S5a-b). Different from the PDAC TME, the SCC TME is mainly composed of malignant epithelial cells, with only a few immune cells infiltrated [29].

We identified 460 known ligand-receptor interactions in sample P2_rep2 (Fig. 1c), with 24 short-range and 24 significant long-range (Additional file 1: Fig. S1c). The ligand-receptor distance distribution was similar to the distribution in the PDAC dataset (Additional file 1: Fig. S4c and S5c), and the peak of the *d* _ *ratio* distribution was closer to 1.0, indicating a spatial interaction tendency toward short-range interactions. For example, the interactions DSG1–DSC3, LAMA3–(ITGA3+ITGB1), and DLL1–NOTCH1 (Additional file 1: Fig. S4d) were all short-range interactions, which rely on the physical contact between ligand and receptor. DSG and DSC were consistently observed to present the colocalization on the apposed cell surfaces in desmosomes and form the basic adhesive unit of desmosomes [38]. LAMA3 binds to its high-affinity receptor and mediates the attachment and migration of cells [39]. Different from short-range interactions, long-range interactions have distinct spatial distributions. For example, for the FGF7–FGFR2 interaction, the FGF7 was mainly expressed in the upper section of the malignant cells, while the FGFR2 was expressed in the bottom section, indicating a long-range interaction between different malignant cells (Additional file 1: Fig. S5d). We found 3 near cell type pairs and 6 far cell type pairs in 15 cell type pairs, which might be caused by the high malignant epithelial proportion in the TME that separates different cell types (Additional file 1: Fig. S5e-f).

We then evaluated the CCI consistency with spatial information. Similar to the results in PDAC, CellChat, ICELLNET, NicheNet, and CellPhoneDB ranked in the top 4 by DES, indicating their stable performance in the TME (Fig. 5b, averaged result using all 3 SCC samples). CellPhoneDB showed the best consistency with the commonly identified interactions in the SCC dataset, with Connectome and ICELLNET ranked as top 2 and 3 (Fig. 5c). These three tools also showed a high rank in the PDAC dataset (Fig. 4b, c). Although the evaluation using DES or using common interactions are stable in the TME, they show different properties of CCI tools, with NicheNet, ICELLNET, and CellChat having better consistency with spatial information, and CellPhoneDB, Connectome showing better overlap with common interactions.

Interestingly, although malignant cells are abundant in the TME according to the ST data, the number of interactions of malignant cells predicted by different tools did not display a comparable dominant fraction (Additional file 1: Fig. S5g, red). The less abundant immune cells and fibroblasts, on the other hand, are involved in a large number of interactions (Additional file 1: Fig. S5g, royal blue, sky blue, and yellow). This could explain that although the overall spatial interaction tendency is toward short-range interactions, we could still identify many long-range interactions (24) since the immune cells and fibroblasts are distributed far away (Additional file 1: Fig. S5e-g). The common interactions identified by different tools increased compared to PDAC datasets, maybe because of a larger number of detected interactions (Fig. 5d). However, for most of the tools, the overlaps with common interactions are still less than 25%, indicating a highly dynamic result between different tools (Fig. 5d). The statistical-based methods still have more consistent interactions with each other, which could be also observed if we focused on the top 10 common interactions (Fig. 5d, e). Taken together, the two datasets from the TME suggest a short-range interaction tendency between different cell types. NicheNet is the best tool that is consistent with spatial information, while statistical-based methods like CellChat and CellPhoneDB showed a balanced performance both on spatial information and consistency with commonly identified interactions.

#### Mouse cortex dataset

**Fig. 6:**
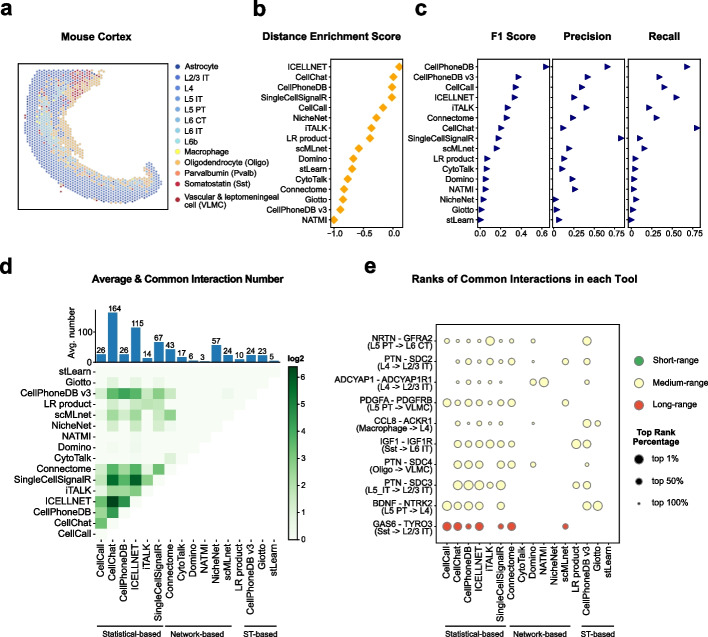
Evaluation results in the mouse cortex dataset. **a** Spatial cell type annotation result of mouse cortex dataset’s ST data. Color indicates the spot’s cell type. **b** The dot plot of tools’ average distance enrichment scores (DES), sorted by the DESs. **c** The dot plots of tools’ average relative accuracy metrics (F1 score, precision, recall), all sorted by the F1 score. **d** The heatmap shows the average common interaction number between each tool’s result per cell type in the mouse cortex dataset. Color displays the interaction number. The bar plot on the top shows the average interaction number predicted by each tool per cell type. The actual number is labeled on the top of each bar. **e** The dot plot of top 10 most common interactions among all tools in the mouse cortex dataset, sorted by interaction’s common count. The dot size indicates the top rank percentage of the interaction in corresponding tool’s result. The dot color reflects interaction’s spatial distance tendency in the mouse cortex dataset. The CCI tools are grouped by their model types

The nervous system is known to have clearly defined layer structures. We applied our workflow to a mouse cortex dataset from the nervous system. After spatial cell type annotation, we identified 13 cell types in ST data (Fig. 6a), including 7 types of glutamatergic cells (L2/3 IT, L4, L5 IT, L5 PT, L6 CT, L6 IT, L6b), 2 types of GABAergic cells (parvalbumin, somatostatin), and 4 types of non-neuronal cells (astrocyte, macrophage, oligodendrocyte, vascular and leptomeningeal cell). The different types of glutamatergic cells were named based on their layer-specific marker and layer enriching dissections [40], which could be successfully reconstructed by the ST data (Fig. 6a, Additional file 1: Fig. S6a-c).

The spatial interaction tendency of ligand-receptor pairs in the mouse cortex dataset showed a long-tail distribution with a preference for long-range interactions, which might be due to the large spot number and the concave shape of the tissue (Additional file 1: Fig. S6d). We observed fewer interactions with a *d* _ *ratio* less than 1 and more interactions with a *d* _ *ratio* larger than 3. We used 723 ligand-receptor interactions with 76 short-range interactions and 76 long-range interactions to evaluate the CCI tools (Additional file 1: Fig. S1c). All three short-range interactions that we showed display spatially colocalized patterns (Additional file 1: Fig. S6e). The neurexin (NRXN) and neuroligin (NLGN) are neuronal cell surface proteins and they can interact with each other to involve in the maturation of glutamatergic and GABAergic synapses [41]. The three long-range interactions were enriched in cell types that have distinct spatial distribution [42, 43, 44]. Using all 13 cell types, we defined 38 near cell type pairs and 9 far cell type pairs according to their relative spatial positions (Additional file 1: Fig. S6f). The nicely organized layer structures lead to the majority of cell types being close to each other, such as L4 and L5 IT, L6b, and L6 IT (Additional file 1: Fig. S6g).

For the mouse cortex dataset, ICELLNET, CellChat, CellPhoneDB, and SingleCellSignalR have better consistency with spatial information evaluated by the DES score (Fig. 6b). This is in general consistent with the results from the TME. However, the comparison with commonly inferred CCIs is not so stable (Fig. 6c). CellCall ranked third by F1 score in the mouse cortex dataset, but in PDAC and SCC datasets, it only ranked at 15 and 11. In summary, the metric of commonly inferred interactions seems to be not as stable as the metric that evaluates the interaction possibility using spatial distance tendencies in different biological systems.

We next compared the interaction patterns between different tools, and the results showed that the cell types of the mouse cortex were highly connected, as reflected by most CCI tools except for CytoTalk, NATMI, Giotto, and stLearn (Additional file 1: Fig. S6h). The different patterns of these tools might be due to the low number of significant CCIs identified by each tool (Fig. 6d, 3 for NATMI, 5 for stLearn, 17 for CytoTalk, 23 for Giotto). Again, although the interaction numbers and patterns are similar between most of the tools, the common interactions were few (Fig. 6d, less than 20% for most pairwise comparisons between tools), and the statistical-based methods have overall better consistency than network-based and ST-based methods (Fig. 6d,e).

#### Human heart dataset

**Fig. 7:**
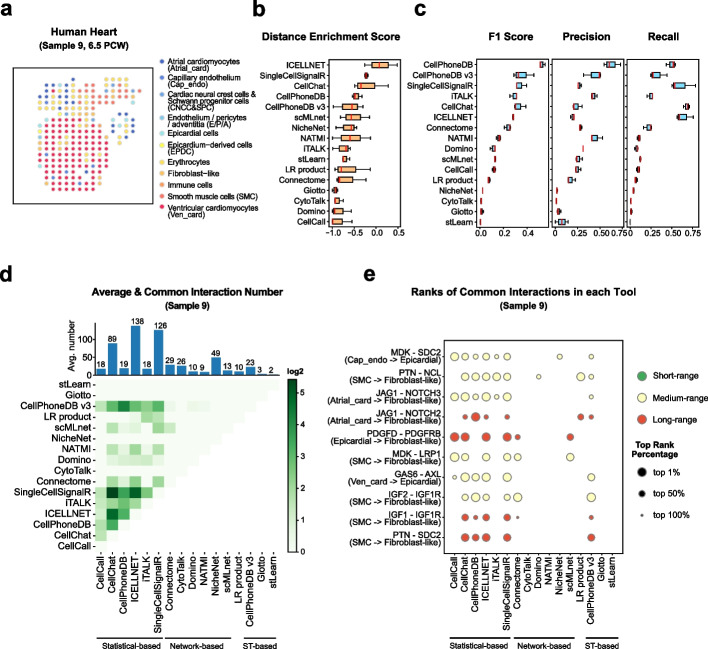
Evaluation results in the human heart dataset. **a** Spatial cell type annotation result of ST data of sample 9 in the human heart dataset. Color indicates the spot’s cell type. **b** The box plot of tools’ average distance enrichment scores (DES) evaluated using all samples (sample 8, sample 9, sample 10) in the human heart dataset, sorted by the median DES. **c** The box plots of tools’ average relative accuracy metrics (F1 score, precision, recall) evaluated using all samples (sample 8, sample 9, sample 10) in the human heart dataset, all sorted by the median F1 score. **d** The heatmap shows the average common interaction number between each tool’s result per cell type in the sample 9. Color displays the interaction number. The bar plot on the top shows the average interaction number predicted by each tool per cell type. The actual number is labeled on the top of each bar. **e** The dot plot of top 10 most common interactions among all tools in the sample 9, sorted by interaction’s common count. The dot size indicates the top rank percentage of the interaction in corresponding tool’s result. The dot color reflects interaction’s spatial distance tendency in the sample 9. The CCI tools are grouped by their model types

The developmental system has cell type-specific transcriptional profiles corresponding to distinct anatomical regions in different developmental stages. We applied our benchmark workflow on a developmental system dataset from 3 human embryonic cardiac samples collected at 6.5 PCW, here using sample 9 for demonstration. We identified 11 cell types after spatial cell type annotation, including two types of cardiomyocytes (atrial cardiomyocytes and ventricular cardiomyocytes), two types of endothelial cells (capillary endothelium and endothelium/pericytes/adventitia), epicardial cells, epicardium-derived cells, erythrocytes, fibroblast-like cells, immune cells, smooth muscle cells, and a merged cell type population for cardiac neural crest cells and Schwann progenitor cells (Fig. 7a, Additional file 1: Fig. S7a-b). The major cell type in the human heart sample 9 was ventricular cardiomyocytes, which were specifically localized in the ventricle regions (Fig. 7a).

The ligand-receptor distance distribution in the human heart dataset was more concentrated than the tumor or neuron systems (Additional file 1: Fig. S7c, Fig. S4-5c, Fig. S6d), which might be caused by the fewer spot number and tighter arrangement of cells in the heart. We identified 255 known interactions in the human heart dataset with 16 short-range interactions and 16 long-range interactions (Additional file 1: Fig. S1c). Consistently, short-range interactions like GDF11–TGFBR1, COL1A2–ITGA9, and HSPG2–DAG1 were spatially colocalized in the tissue (Additional file 1: Fig. S7d), among which COL1A2 and HSPG2 are known to play important roles in cell adhesion [38, 45]. The three long-range interactions shown are all related to secreted functions [42, 46, 47] and their ligands and receptors are spatially distributed away from each other. As for the cell type pairs, we defined 11 near cell type pairs and 3 far cell type pairs (Additional file 1: Fig. S7e-f), which might be due to the highly mixed cell types around the aorta region.

In sample 9 of the human heart dataset, ICELLNET, SingleCellSignalR, CellChat, and CellPhoneDB were top-ranked by the DES, which is similar to their performance in the TME or nervous system, suggesting the robustness in evaluating significant interactions with their spatial distance tendency (Fig. 7b). Furthermore, all the four tools represented better overall consistency with the commonly identified interactions, but with unbalanced performances in the precision and recall (Fig. 7c). These results suggest that DES and common interaction metrics have similar performance in the heart dataset. Interestingly, CellChat, CellPhoneDB, and ICELLNET also have a better performance in the TME dataset for overlap with common interactions. Considering that the heart data also showed a spatial tendency to short-range interactions like the TME, it might indicate that these tools have a preference for identifying short-range interactions, and the common interaction metrics might be biased by the short interaction distance distributions. In addition, we also observed the generally higher F1 score of statistical-based tools in the human heart dataset which was consistently observed in all the previous datasets. These phenomena highly indicating that the common interaction metrics might not only be biased by the interaction type distributions but also be biased by the tools’ algorithm similarities.

Comparing the interaction patterns between different tools, we did not find a major interacting cell type that is similar to the nervous system, indicating that the cells in the heart were also highly connected with each other (Additional file 1: Fig. S7g). CytoTalk, Giotto, and stLearn showed poor performance with the interactions that existed in limited cell types. Unsurprisingly, the common interactions were still few in the developmental system dataset (Fig. 7d, less than 25%). The top 10 common interactions that we found in the human heart dataset are enriched in the IGF and NOTCH pathways between epicardial cells and atrial cardiomyocytes (Fig. 7e), while these interactions play important roles in atrial development [48, 49, 50]. Different from other datasets, we found 4 long-range interactions in the top 10 common interactions in the human heart dataset (Fig. 7e), which might be caused by the distinct distribution of fibroblast-like cells (Fig. 7a). Besides, statistical-based methods still have overall consistency than network-based and ST-based methods (Fig. 7e).

#### Human intestine dataset

**Fig. 8:**
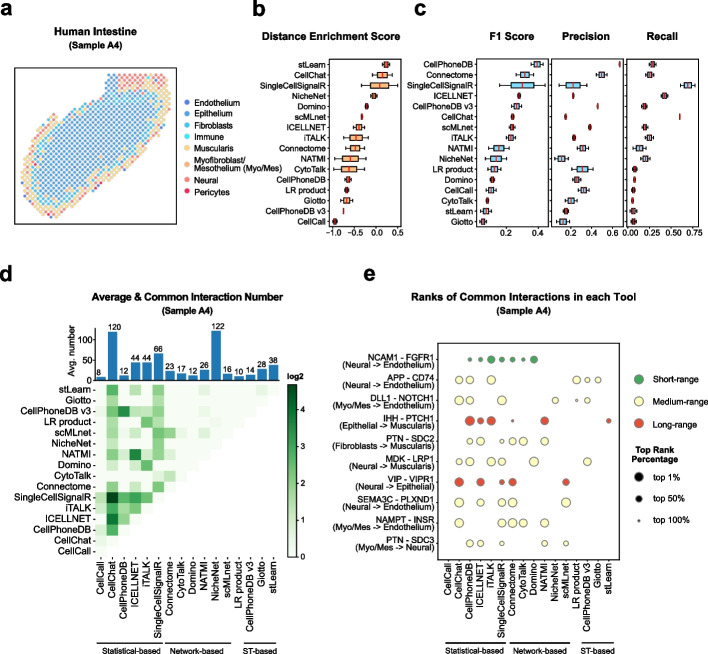
Evaluation results in the human intestine dataset. **a** Spatial cell type annotation result of ST data of sample A4 in the human intestine dataset. Color indicates the spot’s cell type. **b** The box plot of tools’ average distance enrichment scores (DES) evaluated using all samples (sample A3, sample A4) in the human intestine dataset, sorted by the median DES. **c** The box plots of tools’ average relative accuracy metrics (F1 score, precision, recall) evaluated using all samples (sample A3, sample A4) in the human intestine dataset, all sorted by the median F1 score. **d** The heatmap shows the average common interaction number between each tool’s result per cell type in the sample A4. Color displays the interaction number. The bar plot on the top shows the average interaction number predicted by each tool per cell type. The actual number is labeled on the top of each bar. **e** The dot plot of top 10 most common interactions among all tools in the sample A4, sorted by interaction’s common count. The dot size indicates the top rank percentage of the interaction in corresponding tool’s result. The dot color reflects interaction’s spatial distance tendency in the sample A4. The CCI tools are grouped by their model types

Next, we applied our benchmark workflow to another developmental system dataset from human fetal intestine samples collected at 12 and 19 PCW, here using sample A4 for demonstration. We identified 8 cell types in ST data, including endothelium, epithelium, fibroblasts, immune cells, muscularis, neural cells, pericytes, and a merged cluster of myofibroblast and mesothelium (Fig. 8a, Additional file 1: Fig. S8a-c). Cell types in this human intestine sample represented a clear layer structure of epitheliums surrounded by the layers of fibroblasts, muscularis, and neural cells successively [32]. The innermost layer (Epithelium) is huge and occupied most of the slides.

Interestingly, although from the tissue architecture, many cell types were spatially closed due to the layer structure. The spatial *d* _ *ratio* density in the human intestine dataset represented a right-tail distribution, with many interactions toward the long-range type (Additional file 1: Fig. S8d). We identified 420 interactions with 23 short-range interactions and 23 long-ranged interactions (Additional file 1: Fig. S1c). The three short-range interaction examples are associated with cell adhesion [51, 52] and displayed spatial colocalized patterns, while the three long-range interaction examples are enriched in spatially distinct cell types (Additional file 1: Fig. S8e) with the function of secreted signaling pathways [43, 53, 54]. Similar to the nervous system, we identified more cell type pairs as near cell type pairs (8) and fewer cell type pairs as long ones (2) due to the layer structure (Additional file 1: Fig. S8f-g).

In the human intestine dataset, stLearn, CellChat, SingleCellSignalR, and NicheNet were top-ranked again evaluated by the DES, but CellPhoneDB showed a relatively poor performance (Fig. 8b). For the metric of commonly inferred interactions, CellPhoneDB, CellPhoneDB v3, and ICELLNET showed the overall highest result similarities, a result similar to the heart system and the TME (Fig. 8c, Fig. 7c, Figs. 4 and 5c).

Comparing the overall interaction patterns between different tools, we found that fibroblasts, myofibroblast/mesothelium, and muscularis were the major interacting cell types in the intestinal development at the 19 PCW stage (Additional file 1: Fig. S8h, sky blue, orange, and yellow), consistent with the findings in the original publication [32]. The overlaps between different datasets were still low and inferred results varied largely across different tools, while statistical-based tools shared more common interactions than network-based and ST-based tools (Fig. 8d,e). To summarize, in the two developmental system datasets, CellChat displayed the best consistency with the spatial tendencies of known interactions, while CellChat, ICELLNET, and CellPhoneDB kept their balanced performances in both spatial distance tendency and overlap with common interactions similar to the TME dataset.

### Distance enrichment score rank

**Fig. 9:**
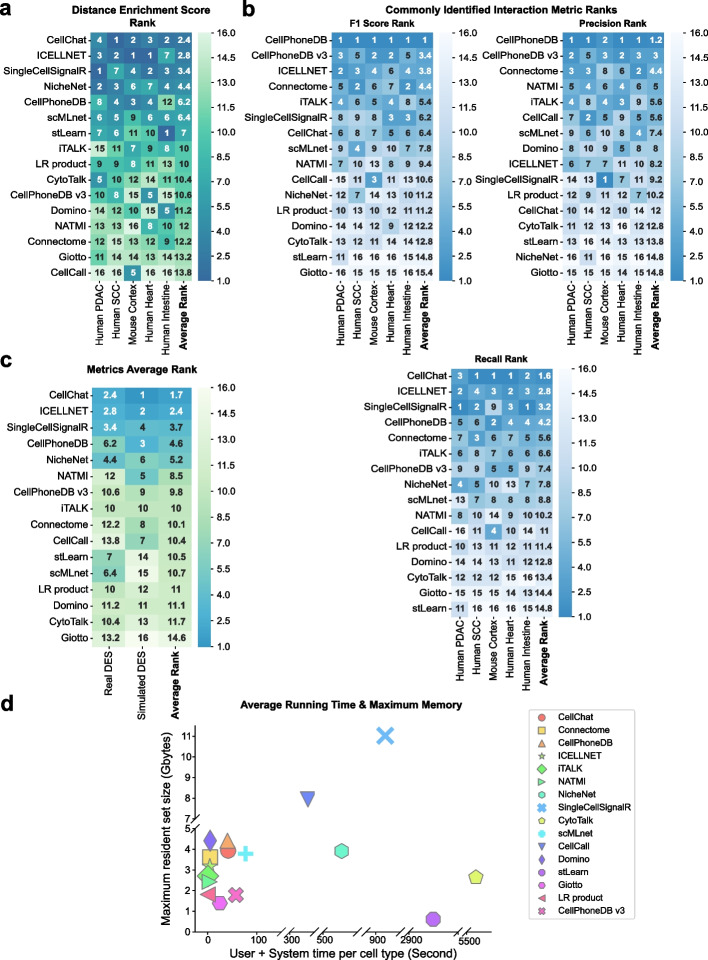
Comprehensive evaluation results. **a** The heatmap shows the rank of each tool’s distance enrichment score (DES) in all 5 datasets and the average DES rank. Tools are sorted by the average DES rank. The actual rank for each tool in each dataset is labeled on the corresponding location. **b** The rank heatmaps of 3 relative accuracy metrics (left: F1 score; medium: precision; right: recall). Tools in each heatmap are ordered by corresponding metric. The actual ranks are labeled on the heatmap. **c** The average rank of tools’ DES in simulated and real dataset. Tools are ordered by the average ranks. The actual ranks are labeled on the heatmap. **d** The scatter plot of average running time and maximum memory usage of each tool in all dataset. The point shape indicates different tools. The average running time of each tool in each dataset is computed by summing up user and system time and then dividing it by the cell type number in the corresponding dataset

We computed the average DES among all 5 datasets to evaluate the overall consistency with spatial information of each CCI tool. CellChat, ICELLNET, SingleCellSignalR, and NicheNet were top-ranked with an averaged DES rank of less than 5 (Fig. 9a), indicating that these four tools almost achieved top 5 performance in every dataset. Among these well-performed tools, CellChat has the most stable performance than the others, which ranked top 4 in all 5 datasets (Fig. 9a). Interestingly, NicheNet has the best consistency with spatial information in two tumor datasets, but the accuracy drops in the nervous or developmental system. One probable explanation is that the prior literature-based network model used in NicheNet has a bias toward gene regulations and cell-cell interactions in the TME. Although the DES ranks of different tools had slight fluctuations in several datasets, the overall DES ranks were still stable across different datasets and systems, especially for ranks of well-performed tools (Fig. 9a). CytoTalk provided a similar way to benchmark CCI tools based on mutual information by integrating scRNA-seq and ST data, and we found CytoTalk evaluation method gave out a similar result to our DES evaluation method. CellChat, NicheNet, and SingleCellSignalR were consistently ranked in the top 5 no matter evaluated using the CytoTalk metric or our DES metric (Additional file 1: Fig. S9a-b, comparing on the human intestine dataset). In summary, the DES metric representing the consistency with spatial information is a stable metric to evaluate the possibility of CCIs, and CellChat has overall the best performance in coordinating with spatial distance tendencies.

### Commonly identified interaction metric ranks

Ranks by the metric of commonly inferred interactions slightly differed from the DES ranks, and several tools with good performance evaluated by DES ranks, such as NicheNet and stLearn, showed a worse performance evaluated by F1 score (Fig. 9b, F1 score rank). CellPhoneDB, CellPhoneDB v3, ICELLNET, Connectome have an average rank of top 5 in the F1 score (Fig. 9b, F1 score rank). Only CellPhoneDB maintained high ranks in both precision and recall metrics within these tools, the other 4 tools all displayed imbalanced performance between precision and recall ranks (Fig. 9b). For example, CellPhoneDB v3 has the top 2 precision rank, but it shows poor performance in terms of recall (Fig. 9b), which may be due to the relatively low number of significant interactions identified by CellPhoneDB v3 (around 20). SingleCellSignalR has a high recall but low precision (Fig. 9b), which may also impact by a large number of significant interactions (60–150). Consistent with what we observed in common interaction patterns in 5 datasets (Figs. 4, 5, 6, 7, and 8d, e), the statistical-based tools, for example, CellPhoneDB, CellChat, SingleCellSignalR, and ICELLNET, obtained higher ranks than network-based and ST-based tools in both F1 score rank and recall rank, indicating the statistical-based methods are more similar with each other (Fig. 9b). The overall better performances of statistical-based tools on the F1 score indicates that the evaluating results of the commonly identified interaction metrics may be biased. Tools with similar algorithms tend to have better performance while evaluating using commonly identified interaction metrics, such as CellPhoneDB and CellPhoneDB v3 (Fig. 9b). In this case, the commonly identified interaction metrics can only be used to reflect the similarity of results from different tools, they cannot be used as measures of the ground truth to evaluate the accuracy of predicted CCIs.

### Metrics average rank

Finally, we integrated the DES ranks from the real and simulated datasets to assign each tool a comprehensive performance rank. CellChat ranked first with an average rank of 1.7, followed by ICELLNET, SingleCellSignalR, CellPhoneDB, and NicheNet (Fig. 9c). CellChat obtained high DES ranks in both real and simulated datasets (2.4 and 1 respectively), indicating its best performances and robustness across datasets and biological systems. CellPhoneDB displayed better consistency with spatial information in the simulated datasets than in the real datasets, possibly due to the statistical-based tools are easier to find significant interactions based on the overexpressed L-R signals (Fig. 9c). Interestingly, ST-based tools, such as Giotto, CellPhoneDB v3, and stLearn, did not get significantly higher ranks than the scRNA-seq-based tools for the DES metric (Fig. 9c). It could be probably caused by the difference in integrating spatial information of these tools and our DES metric. Finally, the top 5 ranked tools indicated an overall better performance of statistical-based tools than network-based tools, with four statistical-based (CellChat, SingleCellSignalR, CellPhoneDB, ICELLNET) and only one network-based (NicheNet) (Fig. 9c).

### Running time and maximum memory usage

In addition to the metrics of DES and commonly inferred interactions, we also compared the average running time and maximum memory usage for each tool (see “Methods”). We found that most tools had similar running times and maximum memory usage which clustered at the bottom left (11 out of 16), most of which (4 out of 11) are statistical-based tools (Fig. 9d). Apart from them, NicheNet, CytoTalk, SingleCellSignalR, and stLearn had higher running time and maximum memory usage (Fig. 9d). CytoTalk is originally designed for constructing de novo intracellular and intercellular signaling networks rather than simply predicting CCIs. For that reason, CytoTalk starts its workflow by generating two intracellular signaling networks between two cell types, which is quite time-consuming. Thus, CytoTalk may not an efficient tool for predicting CCIs, but it can still be a good choice for exploring the intracellular signal transduction network between two cell types. NicheNet, another network-based tool, needs to evaluate the activity of ligands, targets, and receptors from the expression matrix and prior signaling model, respectively, which need much more time than just focusing on ligands and receptors. Though stLearn had a high running time because of its permutation step, ST-based tools tend to have less running time and maximum memory usage than scRNA-seq-based tools since the ST data has a relatively smaller size than scRNA-seq data (Fig. 9d). Most statistical-based tools only focused on the expression of ligands and receptors, so the main time costs are from the iterations in the statistical test step. In this case, they are less time-consuming and have less memory usage than those network-based tools (if the permutation times in statistical tests are not too many). In this case, although SingleCellSignalR can generate relatively robust results, considering the time and memory usage, CellChat and CellPhoneDB are more recommended to estimate CCIs as they will scale well when the cell numbers increased, especially for those consortium projects which usually generate millions of cells.

## Discussion

Cellular crosstalk based on CCIs is the fundamental basis of many biological processes. The increased throughput of scRNA-seq technologies enabled computationally inferring CCIs based on ligand-receptor information. Although numerous CCI tools have been developed, their results are highly dynamic and no ground truth is provided to evaluate the accuracy. Validation of these interactions through loss-of-function experiments or high-throughput screening like CIM-seq [55] is labor-intensive and time-consuming. There is a great need to find reliable and cost-effective benchmarks for evaluating CCIs.

In this study, we integrated the spatial colocalization information with the gene expression for evaluating the possibility of predicted CCIs. We characterized the CCIs into long-range and short-range interactions based on ligand-receptor expression distribution on the ST datasets, and developed a comprehensive workflow to benchmark the performance of CCI tools using consistency with spatial interaction distance and commonly identified interactions. We applied our workflow on 16 different CCI tools using 20 matched ST and scRNA-seq datasets (15 simulated and 5 real datasets). Our results suggest that the DES metric we developed is more stable in evaluating the performance of different CCI tools, while the commonly identified interactions are affected by many factors. Tools that predicted more interactions are more likely to have higher recall ranks, while tools with less predicted results tend to have higher precision. In addition, tools with similar algorithms tend to dominate the common interactions to get higher scores in commonly identified interaction metrics, such as CellPhoneDB and CellPhoneDB v3. Although we tried to use the F1-score to balance the precision and recall, the results are still biased to statistical-based methods, as they tend to have similar interaction predictions and an in general higher overlap. These results suggest that the metric of commonly identified interactions might not be an appropriate strategy for evaluating CCI tools, although it has been widely used in previous algorithm development papers [11, 19, 23]. Finally, the CCIs predicted by different tools vary greatly from each other, with only less than 25% shared pair-wisely for most of the systems we tested. On that basis, the shared interactions by different tools may not be a good standard for evaluating CCI tools, it can only serve as a way to reflect the similarity of results from different tools.

The observed spatial distance tendencies provided useful additional information in our evaluation study, indicating that the combination of multimodal data such as ST and scRNA-seq will improve the performance of CCI predictions. So far, the main restriction on involving ST data in predicting CCIs is that the spatial resolution of currently used ST technologies cannot reach the single-cell resolution. This low spatial resolution will cause a mixture of gene expression patterns of different cell types in a single spot, which heavily influences CCI inference. In this case, scRNA-seq data are still required in these ST-based CCI tools for spatial cell type deconvolution. Recently, some ST technologies with nanoscale resolution have been published, such as Stereo-seq [56] and Seq-Scope [57]. Though the downstream analytic methods for Stereo-seq are still required further development, these high-resolution ST technologies will eventually solve the restriction problem and construct a more accurate spatial CCI environment. We anticipate that the development of CCI tools based on single-cell spatial technology will have higher accuracy in identifying real CCIs than the scRNA-seq-based tools.

Our benchmark suggested that CellChat has a better performance than the other tools, which might be due to CellChat integrating the regulatory information such as the cofactors with ligand-receptor interactions. The regulatory information could better simulate the actual interacting environment in biological processes, which will give predicted interactions more biological meanings. In addition, integrating regulatory information might compensate for the potential drop-out from scRNA-seq data and enhance the final signals. NicheNet also presented a good consistency with spatial information by integrating signal transduction and gene regulatory interactions with basic ligand-receptor interactions in its prior database. Moreover, NicheNet utilizes the known interaction resources by integrating individual data resources into a weighted knowledge network, highly improving the quality and the confidence level of prior knowledge. The mutual corroboration between different databases can find out highly confident resources and remove the potential bias from prior knowledge [58]. In summary, the regulatory information between different ligand-receptor pairs or within the cells might improve the prediction of CCIs and could be considered in the future development of CCI tools.

Interestingly, we found that the ST-based tools that integrate spatial information, such as CellPhoneDB v3, Giotto, and stLearn, did not get significantly higher ranks than the scRNA-seq-based tools for the DES metric. It could be probably explained by the biases induced by different spatial information integrating methods of these tools. In our evaluation workflow, the spatial distance between each ligand-receptor pair is defined using their expression distribution in the whole ST slides. This kind of distance can reflect the ligand-receptor co-expression from the global slide view and is independent of the potential bias induced by cell type annotation. However, in CellPhoneDB v3, Giotto, and stLearn, the spatial distance of each ligand-receptor pair is defined based on the cell type colocalization patterns, which might be significantly affected by wrong cell type mapping or annotations. In our benchmark workflow, the spatial tendency was evaluated using an enrichment score-based method using relative ranks, which will reduce the potential affluence of a few misannotated cells. In addition, those ST-based methods focus more on the ligand-receptor co-expression in a local region rather than the global slide view, which will also cause differences in defining the short and long-range interactions. For the scRNA-seq-based tools, they do not utilize spatial information thus the potential bias in integrating spatial information is the same for all tools. Therefore, although we also benchmarked the performance of ST-based tools, their poor performance might arise from the difference in integrating spatial information, and we recommend benchmarking the scRNA-seq-based tools using our workflow for getting more reliable results.

It is worth mentioning that the thresholds and parameters used for different tools could significantly affect the results, as they may affluence the number of interactions predicted by different tools. In our study, we used the default thresholds and parameters suggested by different tools, as it is very hard to harmonize the significance between statistical-based methods and network-based methods, also most people tend to use the default parameters in predicting CCIs. Besides the parameters, the interaction database (known ligand-receptor pairs) is another key factor that can greatly influence the final result of a CCI tool, and many tools have very few overlaps between ligands, receptors, and interactions. Currently, to consistently compare the results, we used the interaction database from CellChat and only keep the interactions covered by both scRNA-seq and ST data. Although this method can control the impact of different databases, it also leads to a significant drop in the number of interactions we used. And for those tools with complex database structures such as NicheNet, scMLnet, Connectome, and CellCall, it is hard for us to replace their databases with our curated database and we still use the database from the original methods, which will also introduce some bias to our evaluation results of these tools. Another related evaluation work from Dimitrov et al. [58] also highlighted the issue of significant impacts of CCI databases on the predicted results. In summary, both the CCI tools and database can significantly impact the predicted CCIs, thus a high-quality interaction database will definitely improve the prediction results of CCIs.

We also compared our benchmark work with Dimitrov et al. [58] in detail. The evaluation work from Dimitrov et al. mainly focused on comparing the L-R databases used by different CCI tools and analyzed the usage of different databases in influencing CCI prediction. Their results suggested that the coverage of interactions in different databases is biased toward specific subcellular locations and functional categories which will introduce different biases in CCI predicting. These analyses are complementary to our benchmarking study, which mainly focused on evaluating the accuracy of different CCI prediction tools based on the integration of ST data. Both Dimitrov et al. and our study evaluated the performance of CCI tools by comparing the results with common interactions. We have a similar conclusion that the shared interactions between different CCI tools were relatively low, even after we run different tools using the same L-R database. In our benchmark, we also found that the statistical-based methods and network-based methods show different overlaps with common interactions, with statistical-based methods tend to show more consistent interactions between different methods. Besides, we also used the simulated datasets as well as integrated the spatial information from ST datasets to provide a more comprehensive benchmark for the accuracy of predicted CCIs. In summary, our study has some overlap with Dimitrov et al., but we provided more strategies and comprehensive evaluations on the accuracy of CCI predictions, while Dimitrov et al. are more focused on benchmarking the usage of different CCI databases.

Despite our workflow could benchmark the CCIs efficiently, there are still some aspects to improve. The distance measurements of gene distributions and cell type pairs can be further optimized. We used Wasserstein distance as the distance measurement of gene distributions while average Euclidean distance for cell type pairs in our study. All two measurements evaluate distance on a global scale. Though they worked well throughout the whole evaluation process, there are cases where a local distance might be better. For example, in the human intestine dataset, spots in the center region of the epithelial cluster are distal from any other cell types while spots at the boundary of the epithelial cluster are adjacent to fibroblasts. For that reason, it is reasonable to identify short-range interactions between epithelial and fibroblasts from CCI tools. But due to the distance being calculated globally, the pair of epithelial cluster and fibroblasts cluster is regarded as medium distributed cell type pairs. In the future evaluation, it is better to further split some cell type clusters into mini-clusters according to their local structures and evaluate respectively.

## Conclusions

We present a comprehensive workflow to evaluate the performance of cell-cell interaction methods by integrating scRNA-seq data with spatial information. We found that ligand-receptor-based interactions can be separated into short-range interactions enriched in cell-cell contact and long-range interactions enriched in secreted signaling. Using consistency with spatial cell type distribution and with commonly identified interactions, we benchmarked 16 CCI tools using 15 simulated and 5 real datasets. Our results suggested that CellChat has the best performance in consistencies with spatial information. Moreover, CellChat and CellPhoneDB will generate high-confidence results with scaled computational resources, while SingleCellSignalR showed a good performance but consumes much time and memory. The CCI results from different tools are highly dynamic, with statistical-based methods showing more agreement with each other. We recommended combining results from multiple tools to ensure the accuracy of identified interactions.

Taken together, our work provides a concept to integrate spatial information for evaluating the likelihood of the CCIs. Our benchmark could guide the development of future computational tools in resolving the CCIs using multimodal datasets such as ST and scRNA-seq. Finally, to support the future benchmarking of CCI inference performance, we have packaged the benchmark workflow with detailed documentation and included all the benchmark results in simulated and real data in Github (https://github.com/wanglabtongji/CCI).

## Methods

### Datasets

We collected 5 ST datasets with matched scRNA-seq data to explore the spatial distance tendencies and evaluate CCI tools. These datasets are from 3 different biological systems, the human pancreatic ductal adenocarcinoma dataset [28] and the human squamous cell carcinoma dataset [29] are from the tumor microenvironment system; the mouse cortex dataset [30] is from the nervous system; and the human heart dataset [31] and the human intestine dataset [32] are from the developmental system. The scRNA-seq and ST data preprocessing were done by Seurat V3 [59], and the cell type deconvolution of ST data was done by STRIDE [60].

### Human pancreatic ductal adenocarcinoma (PDAC) dataset

The original publication processed primary PDAC tumor samples from two untreated patients for parallel scRNA-seq and ST analysis [28]. We included two samples (PDAC_A: GSM3036911, PDAC_B: GSM4100724) in our study. The corresponding scRNA-seq and ST data were accessed through Gene Expression Omnibus (GEO) under accession number GSE111672. We annotated the cell types of scRNA-seq data by combining the cell type label and marker genes provided in its data source and publication (Fig. 4a, Additional file 1: Fig. S4a-b, using sample PDAC_A as an example).

### Human squamous cell carcinoma (SCC) dataset

The SCC dataset was generated from a published study on human cutaneous squamous cell carcinoma [29]. The ST and matched scRNA-seq data of patient 2 replicate 2 (P2_rep2: GSM4284317), patient 5 replicate 3 (P5_rep3: GSM4284321), and patient 10 replicate 1 (P10_rep1: GSM4284325) were obtained from its original dataset through GEO under accession number GSE144240 and included in our study as the SCC dataset. We used the level 2 cell type annotation provided in its metadata file and the cell type markers in its publication to annotate the cell types of scRNA-seq data. Consistent with its original publication, we removed the multiples, pilosebaceous, eccrine cells from scRNA-seq data. To guarantee adequate cells for each cell type population, we merged CLEC9A DCs, CD1C DCs, plasmacytoid DCs, AS DCs, and Langerhans cells into DCs cluster and merged macrophages and MDSCs into Mono/Macro cluster (Additional file 1: Fig. S5a-b, using sample P2_rep2 as an example).

### Mouse cortex dataset

The scRNA-seq data of the mouse cortex dataset was obtained from a reference dataset of adult mouse cortical cell taxonomy from the Allen Brain Atlas [61], generated with the SMART-Seq2 protocol. The cell type annotations of scRNA-seq data were directly obtained from its data source (Additional file 1: Fig. S6a). We selected the Adult Mouse Brain (FFPE) dataset from the public dataset resources of 10x Genomics [30] as the ST data of the mouse cortex dataset. After preprocessing and clustering, we extracted the cortex region from the ST data using the corresponding H&E staining image as the segmentation reference (Additional file 1: Fig. S6b-c). We furtherly removed 2 spots in cluster 15 that obviously did not belong to the cortex region (barcode: AGACCCACCGCTGATC-1, GAATAGCATTTAGGGT-1, masked by cross signs in Additional file 1: Fig. S6b). Finally, for both scRNA-seq and ST data, we transformed the mouse gene symbols to human gene symbols using biomaRt [62].

### Human heart dataset

The human heart dataset was generated from a published study [31] on revealing the transcriptional landscape of cell types populating during human embryonic heart development. Filtered ST and scRNA-seq data with cell type annotations were downloaded from the Mendeley Data [63]. The ST data from sample 8 to sample 10 was extracted from the original dataset and included in our human heart dataset, which corresponds to 3 6.5 PCW human embryonic heart samples (Additional file 1: Fig. S7a-b, using sample 9 as an example).

### Human intestine dataset

The human intestine dataset was obtained from a published study [32] about the spatiotemporal analysis of human intestinal development. ST and scRNA-seq data were accessed through GEO under accession numbers GSE158328 (ST) and GSE158702 (scRNA-seq). Two samples (A3: GSM4797918, A4: GSM4797919) were included in our human intestine dataset, corresponding to the sample from 12 PCW and 19 PCW colon tissues respectively. The preprocessing and cell type annotation of scRNA-seq data was performed by MAESTRO [64] using the gene markers provided in its publication and the ST data was subset according to the biggest tissue region in the slide (Additional file 1: Fig. S8a-c, using sample A4 as an example).

### Defining short- and long-range interactions

#### Spatial gene expression distribution distance

Using the physical locations of each spot as the coordinates, the gene expression levels as the values, we constructed spatial gene expression distributions from the ST data. The distance between two genes’ spatial expression distributions can be measured by the Wasserstein distance since both distributions are discrete.

Wasserstein distance is a common-used distance measurement of two distributions, which solves an optimal transport problem to evaluate the distance. In our case, suppose ligand gene expressed in *m* spots and receptor gene expressed in *n* spots of ST data, we generate a matrix *D* ∈ *ℝ*^*m* × *n*^ to record the Euclidean distance between spots with ligand or receptor gene expressed according to their spatial coordinates. Then the ligand and receptor gene expression distributions can be flattened into one-dimensional vectors: *L* ∈ *ℝ*^*m* × 1^ and *R* ∈ *ℝ*^*n* × 1^ while still keeping their spatial distances in matrix *D.* The Wasserstein distance from ligand to receptor can be solved by finding an optimal transport plan *γ*^′^ ∈ *ℝ*^*m* × *n*^ which minimizes the total transport cost. The total transport cost can be defined as the summation of the products of transport value and Euclidean distance between each spot. The optimal transport plan *γ*^′^ can be calculated by the following formula:

1 $${\gamma}^{\hbox{'}}=\underset{\gamma \in \varGamma \left(L,R\right)}{\textrm{argmin}}<\gamma, D>$$

Furthermore, based on the optimal transport plan *γ*^′^, the Wasserstein distance can be computed as follows:

2 $${\displaystyle \begin{array}{c}W\left(L,R\right)=\underset{\gamma \in \varGamma \left(L,R\right)}{\min }<\gamma, D>=<{\gamma}^{\prime },D>\end{array}}$$

In summary, the Wasserstein distance is just the minimum total transport cost between two distributions. Since it is time-consuming to compute the Wasserstein distance in two-dimensional space, we applied the Sinkhorn algorithm to accelerate the computing process. The Sinkhorn algorithm is a regularized version of Wasserstein distance and it computes much faster by estimating the optimal solution through iteration. We implemented it through the sinkhorn2 function in POT v0.7.0 [65] with a *reg* parameter set to 0.001. Due to the addition of entropic regularization terms and iterations, the results of the Sinkhorn algorithm on different orders of distributions will be slightly different. In this case, the final distance between ligand and receptor distributions was defined as the mean of the Wasserstein distances from both directions:

3 $${\displaystyle \begin{array}{c}{W}_{LR}=\left(W\left(L,R\right)+W\left(R,L\right)\right)/2\end{array}}$$

where *W*(*L*, *R*) represents the Wasserstein distances from the ligand gene distribution to the receptor gene distribution and the *W*(*R*, *L*) represents the Wasserstein distances with the direction of receptor gene distribution to ligand gene distribution. The *W*_*LR*_ represents the final Wasserstein distance between ligand and receptor. When applying to the real data, we used sctransform [66] to normalize the count matrix before calculating the Wasserstein metric.

#### Ligand-receptor spatial distance ratio and P-value

Since we applied the Wasserstein distance to quantify the distance between two gene expression distributions, we further defined another variable to reflect the spatial interaction tendency.

For a particular ligand-receptor interaction, the ligand gene expression distribution is denoted as *L* while the receptor gene expression distribution is denoted as *R*. The real Wasserstein distance of this interaction (*W*_*LR*_) is denoted as *d* _ *real* for convenience. The random gene expression distributions of ligand and receptor, denoted as *L*_*r*_ and *R*_*r*_, can be constructed by permuting the coordinates for each spot in *L* and *R*. The Wasserstein distance between these two random distributions (${W}_{L_r{R}_r}$) can be calculated in the same way, denoted as *d* _ *simulation* for convenience. Repeating this permuting process for adequate times (1000 times in our case), a set of *d* _ *simulation* can be generated. In this way, the ratio of the *d* _ *real* to the mean of the *d* _ *simulation* set, denoted as *d* _ *ratio*, can be used to quantify the spatial tendency of this interaction:

4 $${\displaystyle \begin{array}{c}d\_ ratio=\frac{d\_ real}{{~}^{{\sum}_{i=1}^nd\_ simulatio{n}_i}\!\left/ \!{~}_{n}\right.}\end{array}}$$

where *n* is the permuting times. Furthermore, a null distribution of the *d* _ *real* can be constructed through the *d* _ *simulation* set. Then a *P*-value can be obtained by a one-sided permutation test based on it, indicating the significance of the spatial interaction tendency. In our case, both left-sided and right-sided *P*-values are calculated to select the short- and long-range interactions, respectively.

#### Filter short-range and long-range interactions

We ranked interactions by their spatial distance tendencies (*d* _ *ratio*) and significances (*P*-value) to filter short-range or long-range interactions. We first extracted and rearranged ligand-receptor interactions in CellChatDB [11] as the known interactions. CellChatDB assigned each interaction an interaction type annotation, such as cell-cell contact type and secreted signaling type, which is convenient for the functional validation. It needs to be emphasized that the interaction type annotations from CellChatDB do not influence the definition of the following short/long-range interactions, they are only used as an additional reference to validate the accuracy of our definitions. Moreover, CellChatDB contains multi-subunit complexes which can fit the requirement of multi-subunit-included CCI tools, such as CellChat, CellPhoneDB, and ICELLNET. Adapting to those CCI tools which do not consider multi-subunit complexes, we split multi-subunit complexes and rearranged them into single-subunit interactions. The original multi-subunit interactions were kept as well. The union of CellChatDB and those new rearranged single-subunit interactions was then used as the known interaction list in our following steps.

For each ST dataset, we filtered those interactions which both ligand and receptor genes expressed in at least 10% of the total spots from our known interaction list. Since the gene capture rates vary across ST datasets, each dataset has its own filtered interaction pairs. We then calculated *d* _ *ratio* and *P*-value of each filtered interaction and ranked them. For filtering interactions of high spatial tendency, we used flexible thresholds (top 10%) of *d* _ *ratio* and *P*-value since the density distributions of *d* _ *ratio* in different datasets have different means and variations (Additional file 1: Fig. S4-5c, Fig. S6d, Fig. S7c, Fig. S8d). We ranked interactions by their *d* _ *ratio* and left-sided *P*-value in both ascending order and selected the top 10% of ranked interactions as the potential short-range interactions. Then we ranked interactions by their *d* _ *ratio* and right-sided *P*-value in descending order and ascending order, respectively, and selected the top 10% of them as the potential long-range interactions. The remaining interactions were classified as medium-range interactions with no apparent spatial tendency. We next filtered those potential interactions with *P*-value less than 0.01 as the significant short-range and long-range interactions.

#### Functional validation of short-range and long-range interactions

We performed GO enrichment analysis for the functional validation of short-range and long-range interactions. The ligand genes of short-range and long-range interactions were extracted and used as the input gene list of GO analysis respectively. The GO analysis was implemented using topGO v2.40.0 [67], and annotated using org. Hs.eg.db v3.11.4 [68], finally visualized by clusterProfiler v3.16.1 [69].

### Spatial cell type annotation

We used STRIDE [60] to perform the spatial cell type deconvolution for each ST dataset with their matched annotated scRNA-seq data. STRIDE is a topic modeling-based method for accurately decomposing and integrating ST slides. In our previous benchmark work, STRIDE exhibited the overall best performance among other published cell type deconvolution tools, which ensured the accuracy and robustness of the spatial cell type annotation in our study. Based on the cell type proportions inferred by STRIDE, we annotated each spot by the cell type with the biggest proportion in it.

### Defining near and far cell type pairs

#### Spatial distance between two cell types

After spatial cell type annotation, we constructed spatial distributions of each cell type. Spatial cell type distributions only represent the spatial locations of spots of specific cell type. To simplify, we used the average Euclidean distance as the distance function. The formulas are as follows:

$$dis\left( spo{t}_a, spo{t}_b\ \right)=\sqrt{{\left({x}_{spo{t}_a}-{x}_{spo{t}_b}\right)}^2+{\left({y}_{spo{t}_a}-{y}_{spo{t}_b}\ \right)}^2}$$

5 $${\displaystyle \begin{array}{c}c{t}_{distance\left(c{t}_a|c{t}_b\right)}=\left(\frac{\sum_{i=1}^{n_a}\underset{j=1,2,\dots,{n}_b}{\min } dis\left( spo{t}_{ai},\kern0.5em spo{t}_{bj}\right)}{n_a}+\frac{\sum_{j=1}^{n_b}\underset{i=1,2,\dots, {n}_a}{\min } dis\left( spo{t}_{bj},\kern0.5em spo{t}_{ai}\right)}{n_b}\right)/2\end{array}}$$

where *ct*_*a*_ and *ct*_*b*_ stand for the cell type *a* and cell type *b*; *n*_*a*_, *n*_*b*_ are the spot number of corresponding cell type; *spot*_*ai*_ is the *i*th spot in cell type a; function *dis*() computes the Euclidean distance between two spots. Then, the spatial distance between cell types can be quantified through the mean of the minimum Euclidean distances between spots (Additional file 1: Fig. S2a).

#### Near and far cell type pairs

We named the combination of two specific cell types as the cell type pair and classified cell type pairs in each ST sample into three types: near, medium, and far based on their spatial distances. Since spatial distances had different ranges in different samples, it is hard to decide fixed boundaries for clustering spatial distances between cell types. So, we just applied the *k*-means clustering (*k*=3) to partition these cell type pairs into 3 clusters and annotated them as near, medium, and far in the order of their average distances.

### Distance enrichment score

Inspired by the enrichment score (ES) used in gene set enrichment analysis (GSEA) [36], we defined the distance enrichment score (DES) to quantify the consistency between excepted and observed spatial distance tendencies. The higher DES indicates the better consistency between expected and observed spatial tendency. We first ranked the short-range and long-range interactions by their *d* _ *rat* and *P*-value to form the expected ranked interaction lists *L*_*s*_ = {*lr*_1_,  *lr*_2_, …, *lr*_*ns*_} and *L*_*l*_ = {*lr*_1_,  *lr*_2_, …, *lr*_*nl*_}, where the *lr*_*i*_ is the *i*th ligand-receptor interaction, the *ns*, *nl* is the total number of short-range and long-range interactions. In the DES calculation part, to get enough common interactions for benchmark in the PDAC dataset, we increased the cutoff in defining short/long-range interactions to the top 15% to fit for its low gene capture rate.

Then we extracted interactions from the CCI tool’s result and formed the observed interaction list *S* for each cell type pair. We denoted the observed interaction list in near cell type pair *ct*_*n*_ and far cell type pair *ct*_*f*_ as *S*_*n*_ and *S*_*f*_. For *ct*_*n*_, the DES can be computed by adding a weighted *P*-value proportion (*P*_match_) when an interaction exists in the *S*_*n*_ and deducting an unmatched weight (*P*_unmatch_) when an interaction is absent in the *S*_*n*_ while walking down the *L*_*s*_. Similarly, for *ct*_*f*_, the DES can also be computed using *S*_*f*_ and *L*_*l*_. The *P*_match_ and *P*_unmatch_ for the *j*th interaction in *L*_*s*_ are defined as follows:

$${P}_{\textrm{match}}\left({S}_n,j\right)=\sum_{\begin{array}{c}l{r}_j\in {S}_n\\ {}j\le i\end{array}}\frac{1-P\_\textrm{valu}{\textrm{e}}_j\ }{\sum_{l{r}_j\in {S}_n}\left(1-P\_\textrm{valu}{\textrm{e}}_j\right)}$$

6 $${\displaystyle \begin{array}{c}{P}_{\textrm{unmatch}}\left({S}_n,j\right)=\sum\limits_{\begin{array}{c}l{r}_j\notin {S}_n\\ {}j\le i\end{array}}\frac{1}{\left| ns- nm\right|}\end{array}}$$

where *nm* is the total number of matched interactions between *S*_*n*_ and *L*_*s*_. The DES is the maximum deviation of (*P*_match_ − *P*_unmatch_) from 0. For DES in *ct*_*f*_, the *P*_match_ and *P*_unmatch_ are of the same formation.

### The metric of commonly identified interactions

Besides DES, we also evaluated the similarity of results from those CCI tools by their commonly identified interactions. We extracted shared interactions that existed at least 3 times among 10 CCI tools’ results as the alternative positive set. The metrics of commonly identified interactions used in our study were precision, recall, and F1 score:

$$\textrm{Precision}=\frac{TP}{TP+ FP}$$

7 $${\displaystyle \begin{array}{c}\textrm{Recall}=\frac{TP}{TP+ FN}\end{array}}$$

$$F1\ \textrm{score}=\frac{2\times \textrm{Precision}\times \textrm{Recall}}{\textrm{Precision}+\textrm{Recall}}$$

where *TP* stands for the true positives, the number of overlapping interactions between commonly identified interactions and predicted interactions. *FP* stands for the false positive, the number of interactions that are absent in shared interactions but exist in predicted results. *FN* stands for the false negatives, the number of interactions that exist in shared interactions but are absent in predicted results.

### Simulating paired scRNA-seq and ST data with the known L-R pairs

We simulated 15 paired scRNA-seq and ST data with the known overexpressed L-R pairs to evaluate CCI tools’ performances. For each biological system, 5 datasets were simulated based on a sample selected from that system. In the TME system, sample PDAC_A of the human PDAC dataset was selected; in the nervous system, the only sample of mouse cortex was selected; in the developmental system, sample A4 of the human intestine dataset was selected. The simulation workflow is as follows (Fig. 3a):

#### Step 1: Simulation of scRNA-seq and ST data

In each simulation round, 4 cell types in ST data are selected and are assigned with the near/far cell type pair defined in the original ST data (Additional file 1: Fig. S3a, using sample A4 as an example). For each cell type, we randomly selected cells from the same cell type in the original scRNA-seq data, and the number of selected cells is based on the number of spots to ensure each spot has approximately 2~5 cells after mapping scRNA-seq cells to ST. Next, the randomly selected cells will be randomly mapped to each spot according to their cell types and replace the spot’s original expression. To keep the real spatial cell type structure in ST data, we did not change the original coordinates of selected spots.

#### Step 2: Simulation of interacted L-R pairs by semi-synthetic strategy

Then for each near/far cell type pair, 30 interactions are randomly selected, and a semi-synthetic method [37] is applied to scRNA-seq data to replace original expression signals of selected ligand and receptor genes with overexpression signals.

#### Step 3: Filtering simulated interactions to keep short/long-range interaction consistent with the spatial distance of cell type pairs

Having simulated ST data, short/long-range interactions can be defined following the same procedure used in the real data. Finally, both simulated scRNA-seq and ST data will be re-modified, only overexpression signals of short-range interactions will be kept in the near cell type pairs, and the same for long-range interactions in the far cell type pairs. After filtering, around 10 simulated interactions will be kept in each cell type pair (Additional file 1: Fig. S3b, using sample A4 as an example). For the kept simulated interactions, if available, corresponding transcription factors and target genes will also be selected and overexpressed so that the tools based on the transcription factors and targets could be also used.

### Make consistent of CCI tools’ databases

To ensure the comparability of each tool’s result, we made consistent of CCI tools’ ligand-receptor databases since the unique interactions in different databases will introduce the bias (Additional file 1: Fig. S2b). CellChatDB, the database of CellChat, contains both regulatory information and multi-subunit complexes which fits most of tools’ requirements, so we chose it to make consistent of other CCI tools’ databases. For some tools (CellCall, Connectome, NicheNet, scMLnet) with a more complex database, we kept their original databases, and only selected interactions common in CellChatDB from their results for evaluation, since collecting and integrating additional information besides L-R pairs is quite time-consuming. For the rest of the tools, we simply replaced their original databases by the CellChatDB to guarantee the comparability between results.

### CCI tools included in this study

We evaluated 16 CCI tools in our benchmark study, including 6 statistical-based tools (CellCall, CellChat, CellPhoneDB, ICELLNET, iTALK, SingleCellSignalR), 6 network-based tools (Connectome, Domino, CytoTalk, NATMI, NicheNet, scMLnet), 3 ST-based tools (CellPhoneDB v3, Giotto, stLearn), and a baseline method. For the choice of parameters for each tool, we will use the default or recommended values normally. If no default or recommended values are supplied, we will try to adjust several parameters to make these tools have similar numbers of predicted interaction numbers with other tools.

#### CellCall

CellCall is a statistical-based tool which also embedded pathway activity analysis in selecting significant interactions. The version of CellCall in our study is 0.0.0.9000, running with its original database and filtering interactions using 0.05 as its *P*-value threshold.

#### CellChat

CellChat is a statistical-based tool using permutation test to select significant interactions between two cell types. The version of CellChat in our study is 1.0.0 and applied to each dataset with significant *P*-value threshold set to 0.05.

#### CellPhoneDB

CellPhoneDB is a statistical-based tool using permutation to calculate the *P*-value (specificity) of a given interaction between two cell types. We used the CellPhoneDB v2 in our study with CellChatDB ligand-receptor database and set significant *P*-value threshold to 0.05.

#### CellPhoneDB v3

CellPhoneDB v3 is a ST-based tool and is the updated version of CellPhoneDB, it will only consider interactions between cell types in the same spatial microenvironment. We defined the spatial microenvironments using cell2location [70] according to its tutorial and selected the significant interactions using 0.05 as the significant *P*-value threshold.

#### Connectome

Connectome is a network-based tool. It treats cell parcellations as nodes and L-R interactions as edges to form a complete interaction network. Connectome uses a system-wide Wilcoxon rank sum test to assign each edge a *P*-value to filter significant edges (interactions). We used Connectome v1.0.1 in our study and filtered interactions with positive score.

#### CytoTalk

CytoTalk is a network-based tool. CytoTalk constructs two intracellular signaling networks based on mutual information between genes and integrated them by known ligand-receptor interactions. Then, CytoTalk uses the score prize-collecting Steiner Forest algorithm (PCSFs) to extract an optimal subnetwork from the integrated network. The remaining ligand-receptor interactions in the subnetwork are significant interactions. The version of CytoTalk included in our study is v4.0.11 and with default parameters (GeneFilterCutoff=0.2).

#### Domino

Domino is a network-based tool finding significant interactions based on the global signaling network. The version of Domino included in our study is 0.1.1. We set the threshold of TF *P*-value to 0.001 and the threshold of receptor-TF correlation coefficient to 0.25 according to their default values.

#### Giotto

Giotto is a ST-based tool using spatial L-R co-expression for predicting. The version of Giotto included in our study is 1.0.4. We used 0.01 as the threshold of *P*-value and 0.1 as the threshold of log fold change.

#### ICELLNET

ICELLNET is a statistical-based tool, using Wilcoxon statistical test to find interactions of high global interaction potential between cell types. The version of ICELLNET in our study is 0.99.3. To make ICELLNET have similar numbers of predicted interaction with other tools, we finally selected top 200 most different interactions with communication score larger than 20 in the step of filtering significant interaction, based on the recommendations.

#### iTALK

iTALK is a statistical-based tool. iTALK first finds differentially expressed ligand and receptor genes between different cell types and searches the ligand-receptor interaction database for both ligand and receptor differentially expressed interactions as outputs. iTALK v0.1.0 was included in our study. To make iTALK have similar numbers of predicted interaction numbers with other tools, we ran the iTALK with the top highly expressed gene number set to 50.

#### NATMI

NATMI is a network-based tool. It treats all the ligand-receptor interactions and cell types together as a weighted-directed-multi-edge network where nodes represent cell types and edges represent ligand-receptor interactions. NATMI selects the top-ranked interactions as the confident interactions based on the edge weights. We ran NATMI with default parameters and using 0.1 as the weight cutoff in our study.

#### NicheNet

NicheNet is a network-based tool. It integrates individual data sources covered ligand, receptor, signal transduction, and gene regulatory interactions into weighted networks as a prior model. Then NicheNet optimizes the weight of each data source in the prior model using the model-based parameter optimization. Based on the prior model and gene expression, NicheNet assigns each ligand-target pair a regulatory potential score using the network propagation method and selects highly potential ligand-receptor-target pairs. The version of NicheNet in our study is v1.0.0. We ran NicheNet with its curated ligand-receptor interactions database and used the top 50 activity ligands for selecting highly potential interactions according to its recommended value and the numbers of predicted interaction numbers of other tools.

#### scMLnet

scMLnet is a network-based tool. scMLnet filters interactions by finding overlapping receptors and TFs in the 3 signaling subnetworks (L-R subnetwork, R-TF subnetwork, TF-target subnetwork) defined by prior knowledge and gene expression. The version of scMLnet in our study is 0.1.0. To make scMLnet have the number of predicted interactions larger than 0 in most cases, we set the cutoffs of *P*-value and log fold change both to 0.1.

#### SingleCellSignalR

SingleCellSignalR is a statistical-based tool. It assigns each interaction a score based on global gene expression and estimates an appropriate threshold for these scores by controlling false positive to filter significant interactions. The version of SingleCellSignalR in our study is 1.4.0. We set the score cutoff to 0.6 according to its recommended value.

#### stLearn

stLearn is a ST-based tool predicting interactions based on L-R co-expression and cell type density. The version of stLearn included in our study is 0.4.7. We set the permutation times to 1000 as recommended and filtered interactions with scores larger than 0.

#### LR product

LR product serves as a baseline method, it selected 10 L-R pairs between each cell type in each interacting direction based on the product of L-R expression.

### Running time and maximum memory usage

We recorded the running time and maximum memory usage of each CCI tool in every dataset. We ran these tools on a server with an AMD EPYC 7552 48-Core Processor with 48 cores and 566 GB RAM with the CentOS 7.9 operating system. For tools that supported multiprocessing, we ran them using 4 cores in each dataset, and for tools that did not have multiprocessing function, we just ran them using 1 core. In that case, we recorded both user time and system time of each tool instead of the real time to ignore the differences between single processing and multiprocessing. The user time and system time were recorded by using the “time” command on the Linux shell. Then we summed user and system time and averaged them by the cell type number in each dataset. Eventually, we used the average of running time per cell type among all 5 datasets as the final running time measurement. The maximum memory usages were also recorded by the “time” command as the maximum resident set size in its outputs. We used the mean of the maximum memories in all 5 datasets as the final average maximum memory for each tool.

## Supplementary Information

Additional file 1: Figure S1-S9. It includes all the supplementary figures and legends.

Additional file 2. Review history.

## Data Availability

The scRNA-seq and ST data of the PDAC dataset can be accessed through GEO under accession number GSE111672 [71]. The scRNA-seq and ST data of the SCC dataset can be accessed through GEO under accession number GSE144240 [72]. The scRNA-seq data of the mouse cortex dataset can be downloaded from Allen Brain Atlas (10.1038/nn.4216) [73], or can also be downloaded from Seurat spatial vignette (https://satijalab.org/seurat/articles/spatial_vignette.html). The ST data of cortex dataset can be downloaded from 10x Genomics website (https://www.10xgenomics.com/resources/datasets/adult-mouse-brain-ffpe-1-standard-1-3-0) [74]. The scRNA-seq and ST data of the human heart dataset can be collected from https://www.spatialresearch.org [75]. The scRNA-seq and ST data of the human intestinal dataset can be accessed through GEO under accession numbers GSE158328 (ST) [76] and GSE158702 (scRNA-seq) [77]. The packaged benchmark workflow with detailed documentation and all benchmark results in simulated and real data are available in the GitHub repository (https://github.com/wanglabtongji/CCI) [78] and Zenodo (10.5281/zenodo.7125650) [79]. The source code is released under GPL-3.0 license.
