# Supplementary material for: Evaluation of cell-cell interaction methods by integrating single-cell RNA sequencing data with spatial information
Source: Genome Biol. 2022 Oct 17;23:218. doi: 10.1186/s13059-022-02783-y (PMC9575221; doi:10.1186/s13059-022-02783-y)
Supplement: Supplementary file 1 — Additional file 1: Figure S1-S9. It includes all the supplementary figures and legends. [file 13059_2022_2783_MOESM1_ESM.pdf]

Figure S1

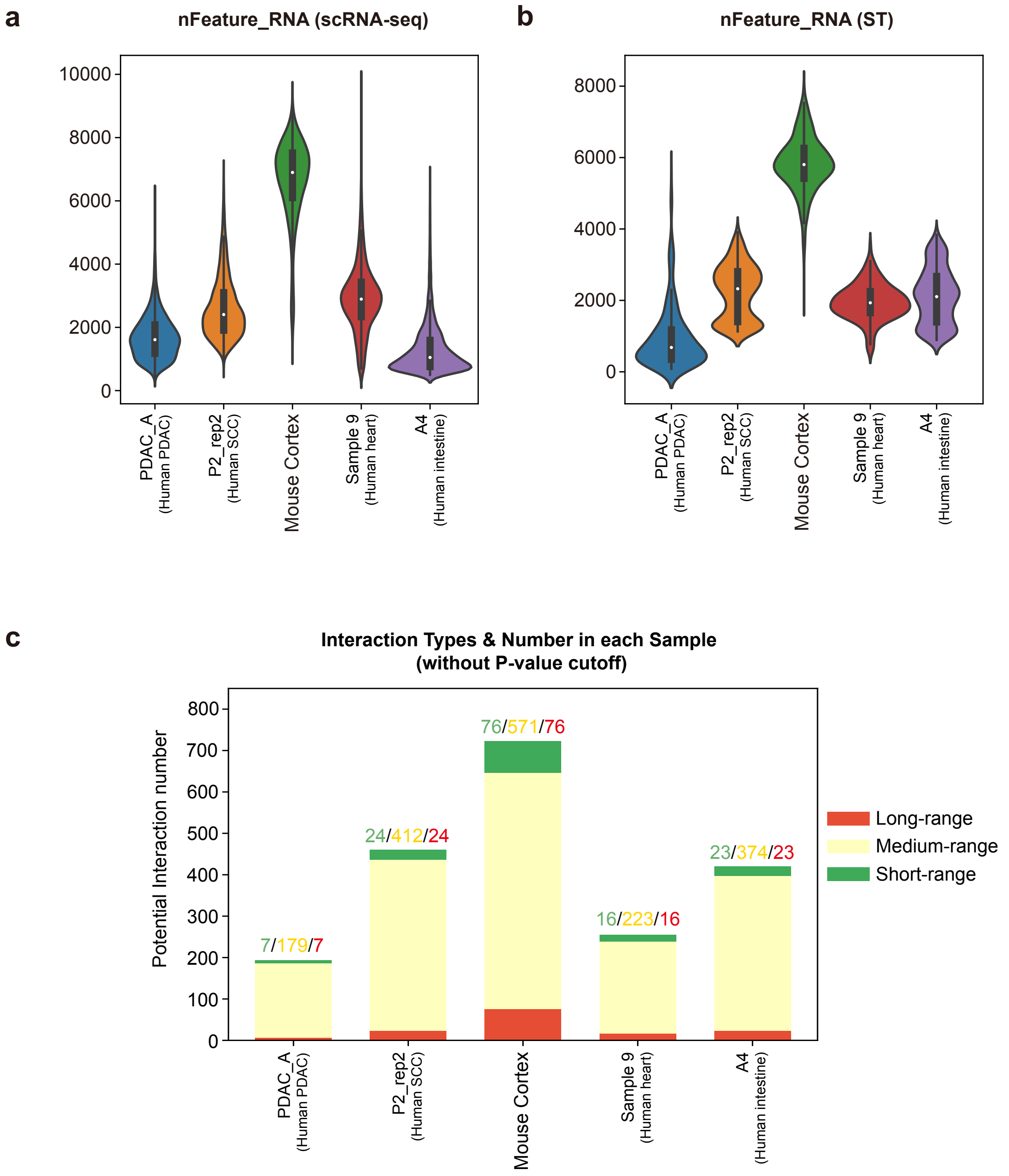

Gene coverages and the actual used short-range and long-range interaction numbers

a. nFeature of exampled samples' scRNA-seq data.

b. nFeature of exampled samples' ST data.

c. The actual used short-range and long-range interaction numbers in calculating distance enrichment score in exampled samples.

Figure S2

a

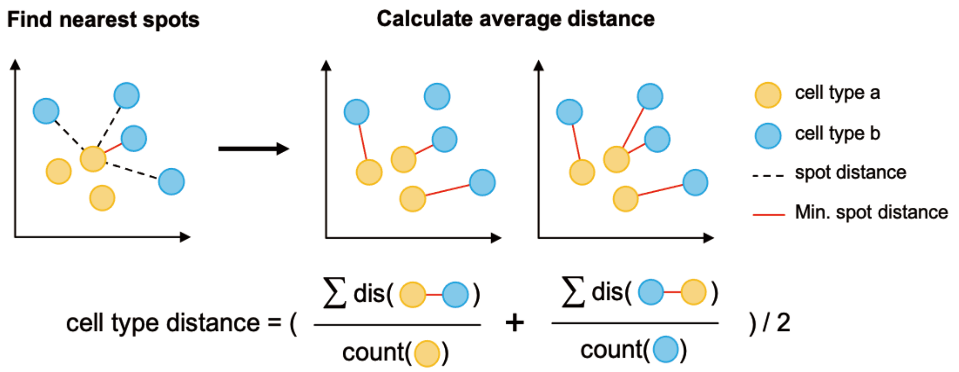

b

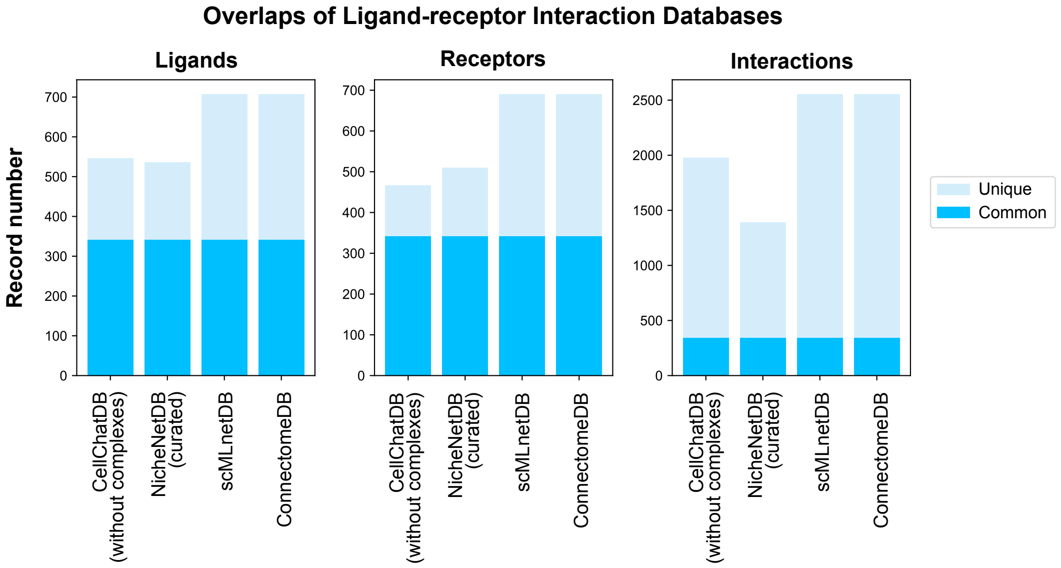

Schematic illustration of computing cell type distance and LR databases overlaps

a. The Schematic figure illustrates the procedure for computing cell type distance. Each point indicates a spot in the ST data, the color represents the cell type. The black dotted line shows the Euclidean distance between connected spots, while the red line indicates that the connected spots have the minimum distance than other black dotted line connected spots. The distance between cell types can be quantified through the mean of minimum Euclidean distances between spots.

b. The bar plots show the overlaps between interaction databases.

Figure S3

a b

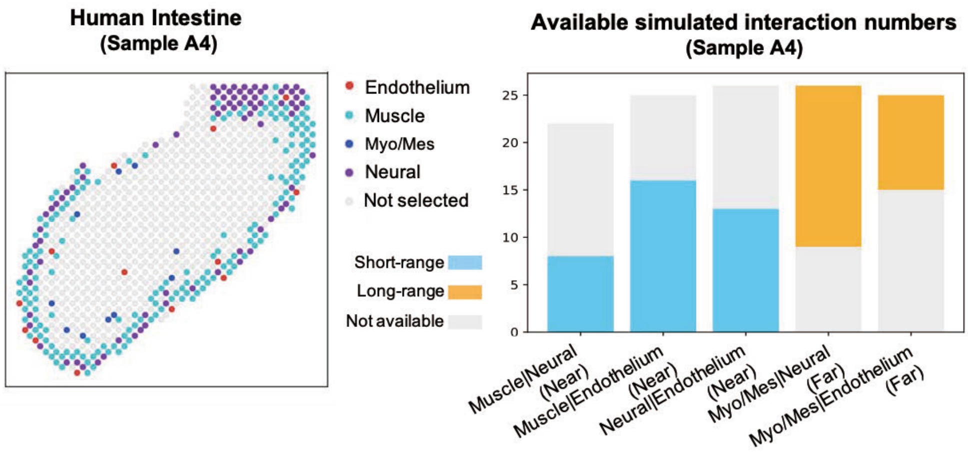

Example of selecting cell types and filtering simulated LR pairs

a. The scatter plot displays the example of randomly selecting 4 cell types in the data simulation procedure. Each point represents a spot in ST data, the selected spots are colored according to their cell types while the unselected spots colored in gray. Using sample A4 as an example.

b. the bar plot shows the number of simulated LR pairs after filtering. The kept LR pairs are colored according to their interaction types. The LR pairs which failed to pass the filtering are colored in gray.

Figure S4

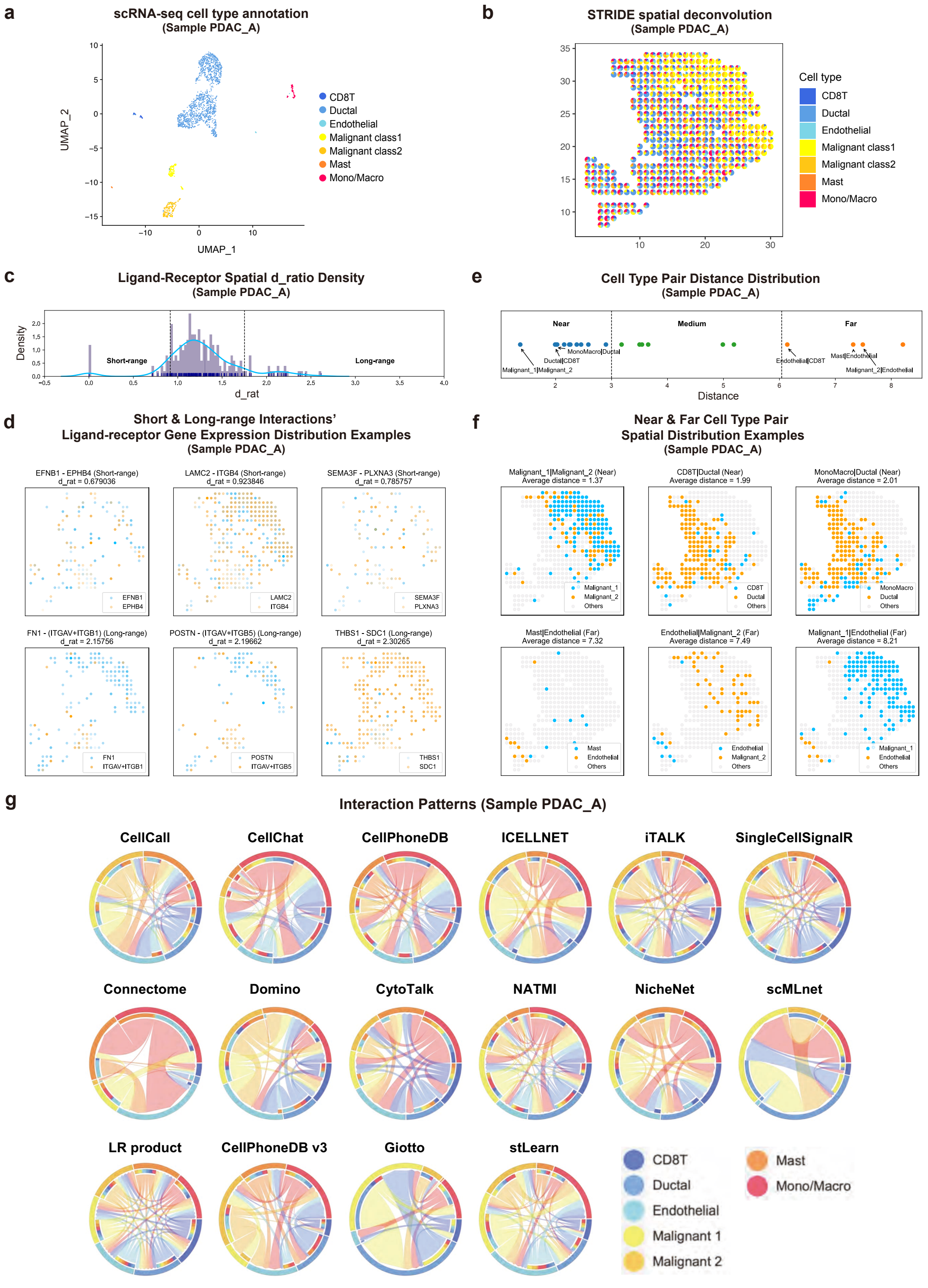

Data processing results and examples of the sample PDAC_A

a. The UMAP of scRNA-seq data of the sample PDAC_A, colored by cell types.

b. The STRIDE deconvolution result of the sample PDAC_A. The pie plot in each spot represents the predicted cell type proportions.

c. The density plot of the *d_ratio* distribution in the sample PDAC_A. The black dotted lines show the *d_ratio* boundaries of short-range and long-range interactions, respectively.

d. The examples of ligands (blue) and receptors' (orange) expression distributions in short-range (top) and long-range (bottom) interactions. The transparency of spot indicates the gene expression level.
e. The dot plot shows the distances of all cell type pairs in the sample PDAC_A, and the clustering results. The dot color stands for the type of cell type pair, blue for near cell type pair; green for medium cell type pair; orange for far cell type pair. The dotted lines are just for better displaying the different groups of cell type pairs and do not stand for the actual distance boundaries.
f. The examples of near (top) and far (bottom) cell type pairs' spatial distributions. The spot color is for dividing cell types.
g. The chord plot of the overall interaction pattern in the sample PDAC_A predicted by each tool. Color indicates cell types. For readability, only cell types in near and far cell type pairs are included in chord plots. The segment size reflects the relative number of interactions that the corresponding cell type participated in. The arrow indicates the interaction direction between cell types and its width shows the relative interaction number.

Figure S5

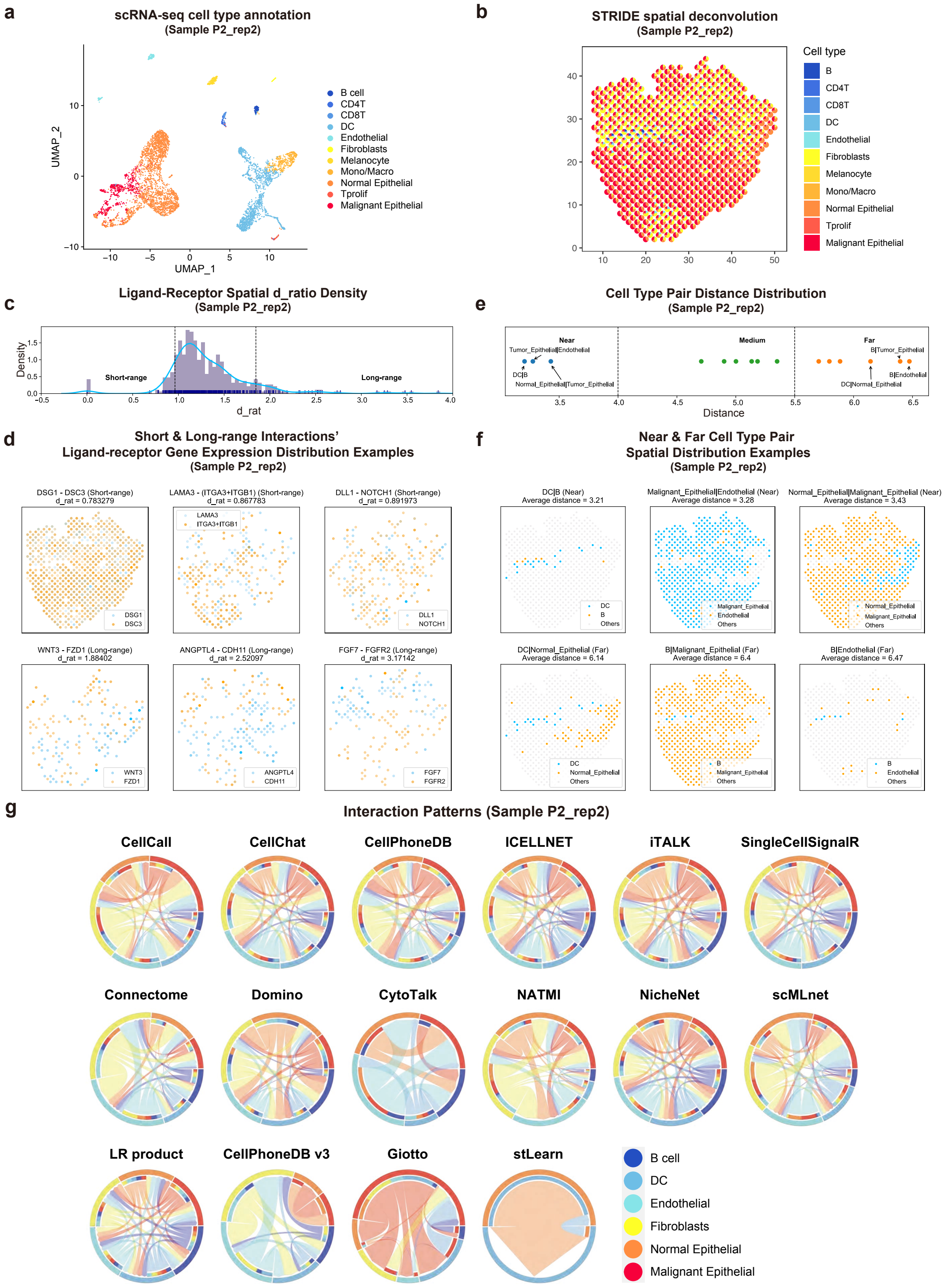

Data processing results and examples of the sample P2_rep2

a. The UMAP of scRNA-seq data of the sample P2_rep2, colored by cell types.

b. The STRIDE deconvolution result of the sample P2_rep2. The pie plot in each spot represents the predicted cell type proportions.

c. The density plot of the *d_ratio* distribution in the sample P2_rep2. The black dotted lines show the *d_ratio* boundaries of short-range and long-range interactions, respectively.

d. The examples of ligands (blue) and receptors' (orange) expression distributions in short-range (top) and long-range (bottom) interactions. The transparency of spot indicates the gene expression level.
e. The dot plot shows the distances of all cell type pairs in the sample P2_rep2, and the clustering results. The dot color stands for the type of cell type pair, blue for near cell type pair; green for medium cell type pair; orange for far cell type pair. The dotted lines are just for better displaying the different groups of cell type pairs and do not stand for the actual distance boundaries.
f. The examples of near (top) and far (bottom) cell type pairs' spatial distributions. The spot color is for dividing cell types.
g. The chord plot of the overall interaction pattern in the sample P2_rep2 predicted by each tool. Color indicates cell types. For readability, only cell types in near and far cell type pairs are included in chord plots. The segment size reflects the relative number of interactions that the corresponding cell type participated in. The arrow indicates the interaction direction between cell types and its width shows the relative interaction number.

Figure S6

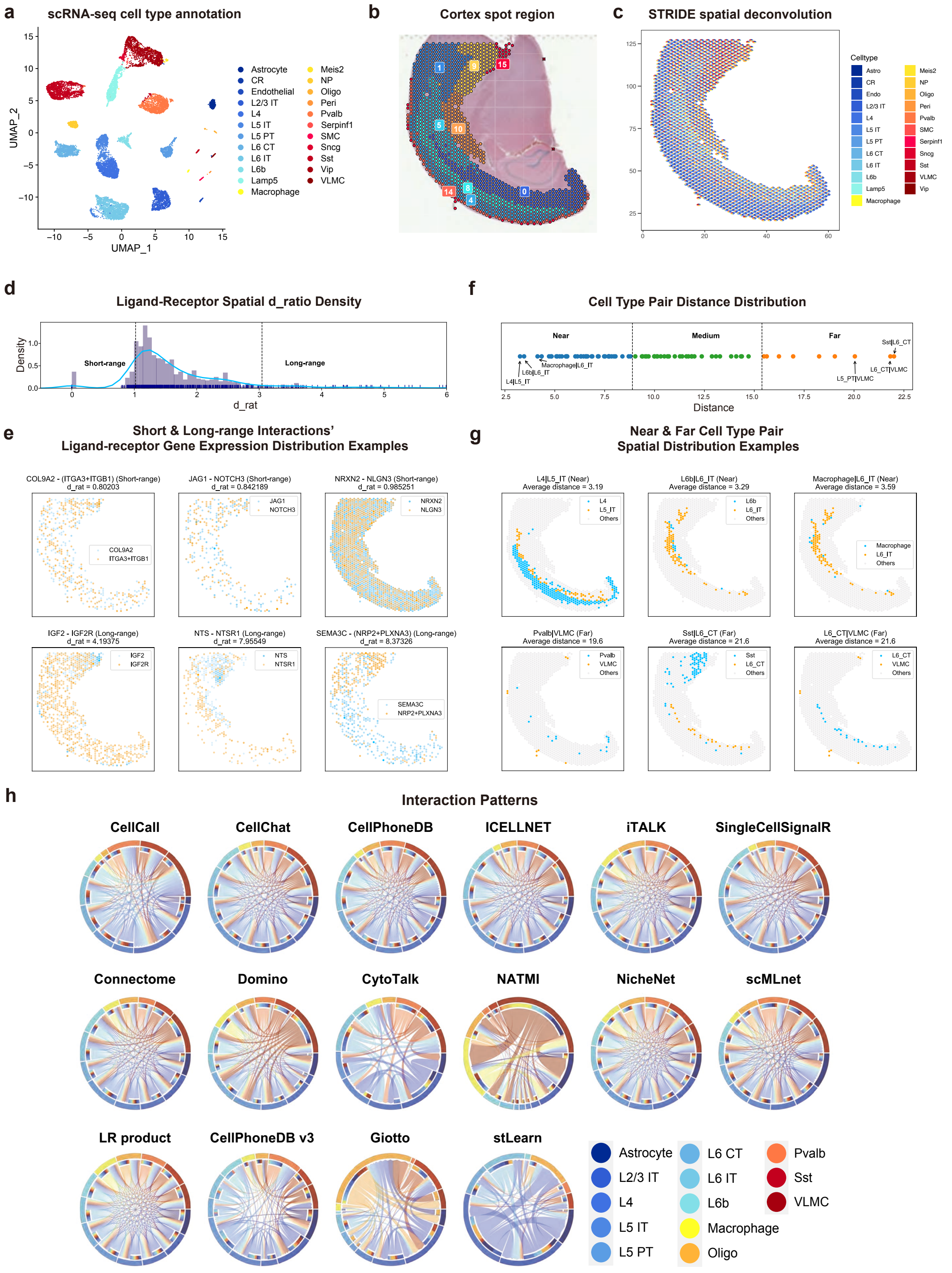

Data processing results and examples of the mouse cortex dataset

a. The UMAP of scRNA-seq data of the mouse cortex dataset, colored by cell types.

b. The spot clusters of the cortex region in the ST data. Numbers and colors for distinguishing different clusters. Two outliers (masked by black cross signs) in the cluster 15 are removed since they are absolutely not in the cortex region.

c. The STRIDE deconvolution result of mouse cortex dataset's ST data. The pie plot in

each spot represents the predicted cell type proportions.
d. The density plot of the *d_ratio* distribution in the mouse cortex dataset. The black dotted lines show the *d_ratio* boundaries of short-range and long-range interactions, respectively.
e. The examples of ligands (blue) and receptors' (orange) expression distributions in short-range (top) and long-range (bottom) interactions. The transparency of spot indicates the gene expression level.
f. The dot plot shows the distances of all cell type pairs in the mouse cortex dataset, and the clustering results. The dot color stands for the type of cell type pair, blue for near cell type pair; green for medium cell type pair; orange for far cell type pair. The dotted lines are just for better displaying the different groups of cell type pairs and do not stand for the actual distance boundaries.
g. The examples of near (top) and far (bottom) cell type pairs' spatial distributions. The spot color is for dividing cell types.
h. The chord plot of the overall interaction pattern in the mouse cortex dataset predicted by each tool. Color indicates cell types. For readability, only cell types in near and far cell type pairs are included in chord plots. The segment size reflects the relative number of interactions that the corresponding cell type participated in. The arrow indicates the interaction direction between cell types and its width shows the relative interaction number.

Figure S7

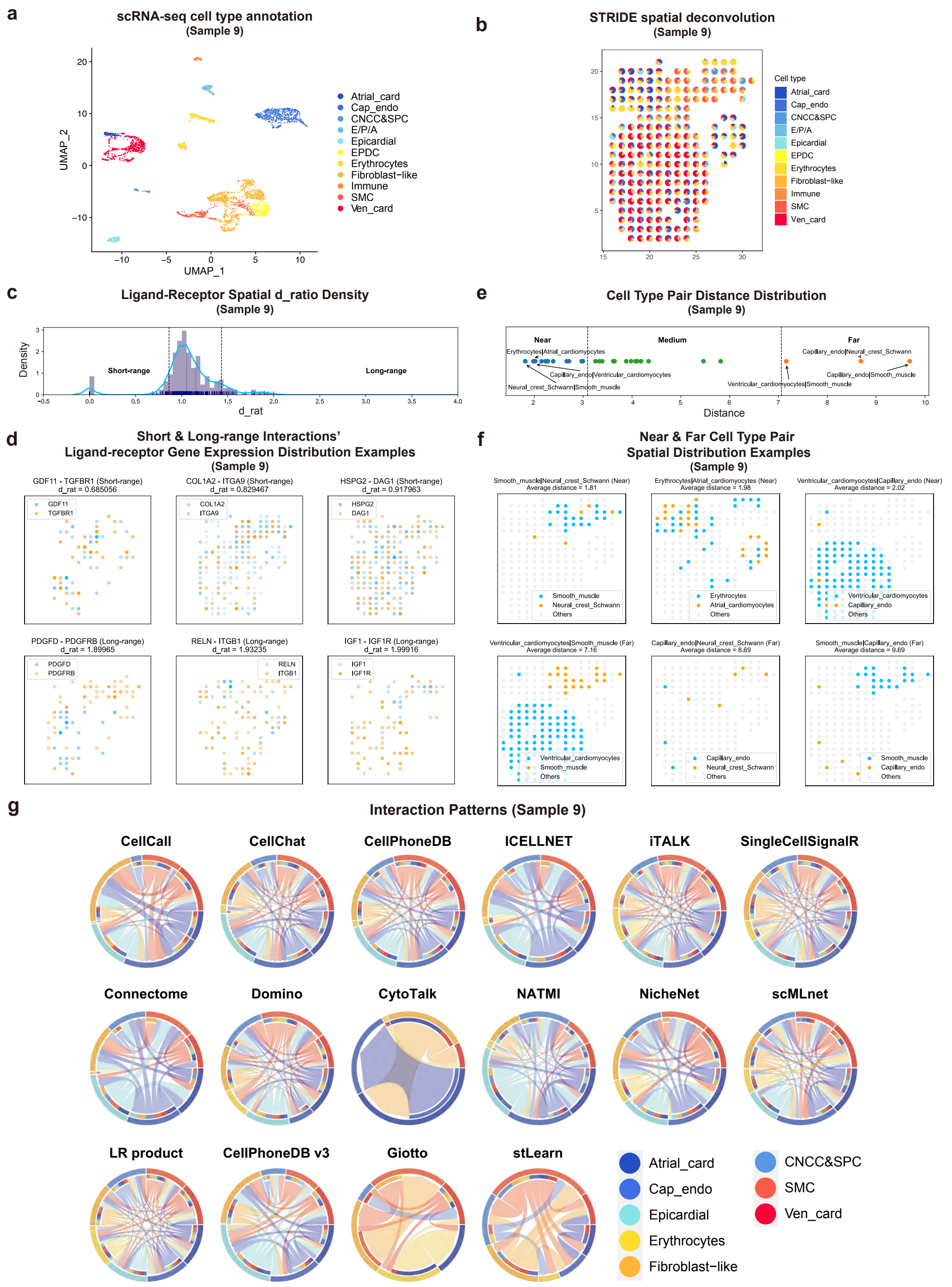

Data processing results and examples of the sample 9

a. The UMAP of scRNA-seq data of the sample 9, colored by cell types.

b. The STRIDE deconvolution result of the sample 9. The pie plot in each spot represents the predicted cell type proportions.

c. The density plot of the *d_ratio* distribution in the sample 9. The black dotted lines show the *d_ratio* boundaries of short-range and long-range interactions, respectively.

d. The examples of ligands (blue) and receptors' (orange) expression distributions in short-range (top) and long-range (bottom) interactions. The transparency of spot indicates the gene expression level.
e. The dot plot shows the distances of all cell type pairs in the sample 9, and the clustering results. The dot color stands for the type of cell type pair, blue for near cell type pair; green for medium cell type pair; orange for far cell type pair. The dotted lines are just for better displaying the different groups of cell type pairs and do not stand for the actual distance boundaries.
f. The examples of near (top) and far (bottom) cell type pairs' spatial distributions. The spot color is for dividing cell types.
g. The chord plot of the overall interaction pattern in the sample 9 predicted by each tool. Color indicates cell types. For readability, only cell types in near and far cell type pairs are included in chord plots. The segment size reflects the relative number of interactions that the corresponding cell type participated in. The arrow indicates the interaction direction between cell types and its width shows the relative interaction number.

Figure S8

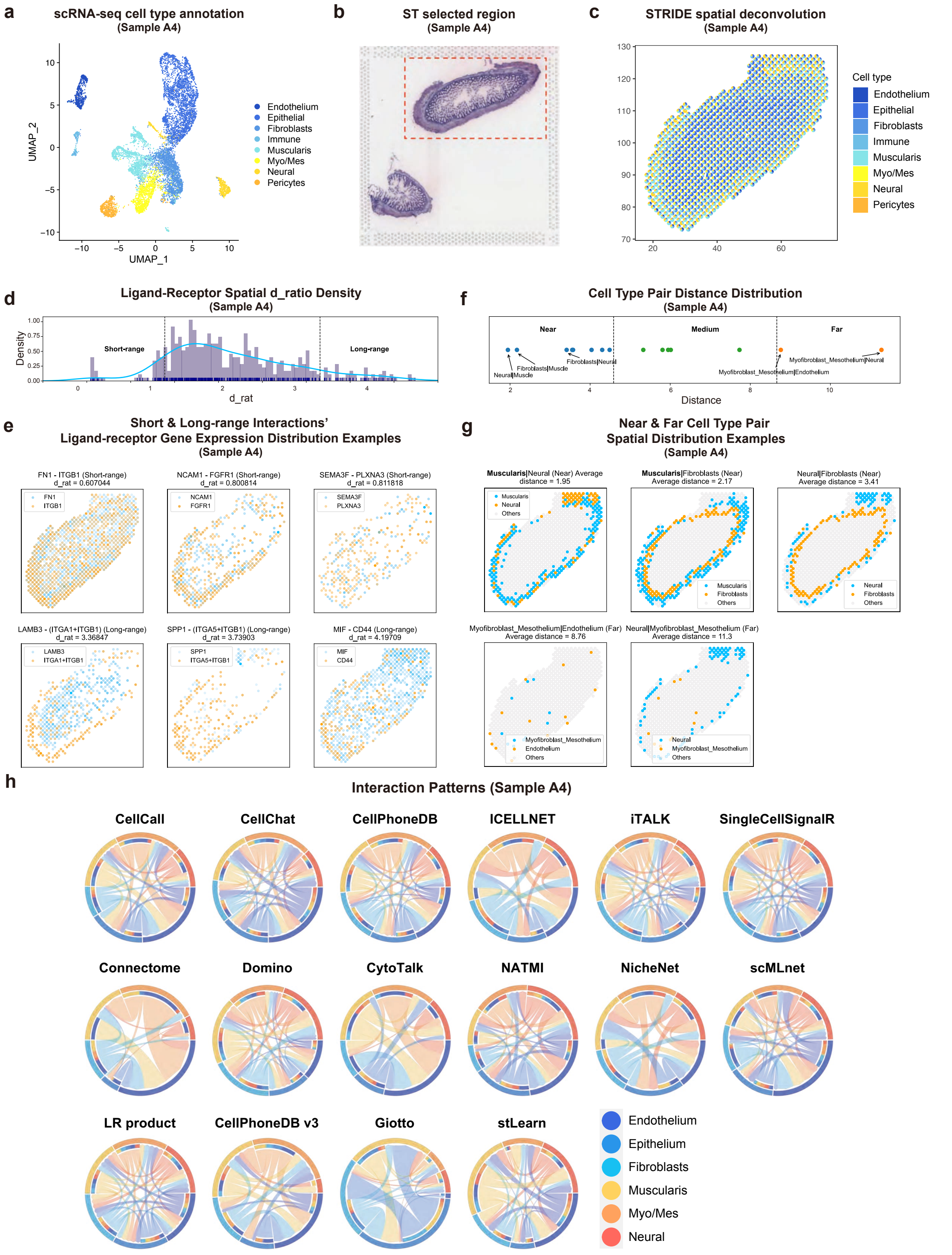

Data processing results and examples of the sample A4

a. The UMAP of scRNA-seq data of the sample A4, colored by cell types.

b. The actually used ST sample region in this study (marked by red dotted line).

c. The STRIDE deconvolution result of the sample A4. The pie plot in each spot represents the predicted cell type proportions.

d. The density plot of the *d_ratio* distribution in the sample A4. The black dotted lines show the *d_ratio* boundaries of short-range and long-range interactions, respectively.
e. The examples of ligands (blue) and receptors' (orange) expression distributions in short-range (top) and long-range (bottom) interactions. The transparency of spot indicates the gene expression level.
f. The dot plot shows the distances of all cell type pairs in the sample A4, and the clustering results. The dot color stands for the type of cell type pair, blue for near cell type pair; green for medium cell type pair; orange for far cell type pair. The dotted lines are just for better displaying the different groups of cell type pairs and do not stand for the actual distance boundaries.
g. The examples of near (top) and far (bottom) cell type pairs' spatial distributions. The spot color is for dividing cell types.
h. The chord plot of the overall interaction pattern in the sample A4 predicted by each tool. Color indicates cell types. For readability, only cell types in near and far cell type pairs are included in chord plots. The segment size reflects the relative number of interactions that the corresponding cell type participated in. The arrow indicates the interaction direction between cell types and its width shows the relative interaction number.

Figure S9

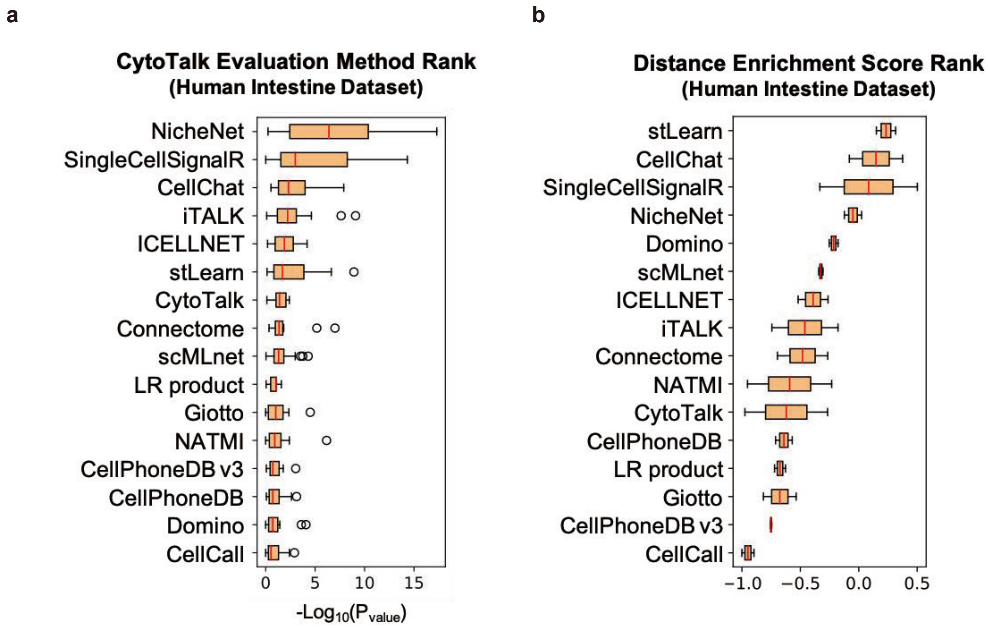

Evaluation results of the CytoTalk and DES metrics are similar.

a. Evaluation rank in human intestinal dataset using CytoTalk evaluation method. The box reflects the overall statistical significance across all samples and cell types. Tools are sorted by the median statistical significance. The ‘LR product’ represents the baseline method based on the product of ligand and receptor expressions.

b. DES rank in the human intestinal dataset. The box plot reflects the overall distribution of DES across all samples. Tools are sorted by the median DES. The ‘LR product’ represents the baseline methxsod based on the product of ligand and receptor expressions.
