# Supplementary material for: Evaluation of cell-cell interaction methods by integrating single-cell RNA sequencing data with spatial information
Source: Genome Biol. 2022 Oct 17;23:218. doi: 10.1186/s13059-022-02783-y (PMC9575221; doi:10.1186/s13059-022-02783-y)
Supplement: Supplementary file 2 — Additional file 2. Review history. [file 13059_2022_2783_MOESM2_ESM.docx]

Review History

**First round of review**

**Reviewer 1**

**Were you able to assess all statistics in the manuscript, including the appropriateness of statistical tests used?**

Yes, and I have assessed the statistics in my report.

**Comments to author:**

Given that increasing attention has been drawn to cell-cell communication analysis in the field of single-cell biology, sysmatical evaluation of computational tools for inferring cell-cell interactions (CCIs) is highly needed. In this manuscript, Liu et al. presented an evaluation framework for systematically benchmarking existing CCI tools. Due to the lack of ground truth, the key assumption of this proposed framework is that spatial distance suggests the interaction tendency of different cell types. The authors proposed a new metric called distance enrichment score (DES) to quantify the coherence between expected and observed spatial distance tendencies by integrating scRNA-seq and spatial transcriptomics (ST) data. After benchmarking 10 cell-cell interaction methods in five matched scRNA-seq and ST datasets, the authors provided suggestions of tools with better performance.

This study provides a timely evaluation framework as well as the guildance for the use of CCI tools, which is valuable to the community of cell-cell communication analysis. However, the current study lacks comparisons with several other tools and also existing tools designed for ST data, which is very important for the guildance of future study. Below is a list of specific comments.

Major comments:

1. Several other tools designed for scRNA-seq were missing in the benchmarking, such as scConnect (Bioinformatics, 2021), CellCall (NAR, 2021) and Dmonino (bioRxiv 2020.07.24.21853). A baseline method based on the product of ligand and receptor expressions should be also included. In addition, it is of great value to compare the performance against the recently developed tools for ST data, such as CellPhoneDB V3, Giotto (Genome Biology, 2021), Cell2Cell (bioRxiv 2020.11.22.392217) and stLearn (bioRxiv 2020.05.31.125658).

2. In the ST data with predicted cell types, most of the cell types had very few number of cells (less than five in some cases). Since the cell labels were computationally predicted from scRNA-seq data, it is important to evaluate the robustness of the predicted cell labels when performing benchmarking. Do inaacurate annotations affect the calculated DES? Since some predicted cell types have extremely small number of cells in several tested datasets, does this affect the calculated DES?

3. The authors only selected one patient sample from ST data when integrating each scRNA-seq dataset. Due to the high variability of patient samples, it is necessary to examine the performance using different patient samples for the same biological system. Does the choice of patients affect the consistency?

4. CytoTalk (Science Advances, 2021) provided a similar way to benchmark CCI tools by integrating scRNA-seq and ST data. It is valuable to include the evaluation method used in this paper.

5. While the authors provided a well-documented evaluation framework, it is worth providing a complete example pipeline for facilitating the comparison analysis when new tools are developed in the future. The authors should (1) provide a documented guildance when new tools are adding into the comparison analysis; and (2) provide the benchmarking codes for all tested datasets when the scRNA-seq data and cell labels of ST data are given.

Minor comments:

1. Is there any reason why NicheNet performs well in two tumor datasets but not in the other two datasets?

2. Is there any reason why the authors used the top 50 ligands in NicheNet but the top 100 in ICELLNET? In scMLnet, the authors metioned "To control the predicted interaction number". Can the authors add more details on the criteria of the choice of parameters for selecting inferred interactions in different methods.

3. In line 705, the authors used the top 10% to define the long and short range of interactions, but used the top 15% in line 844. Why not use the same parameter?

4. Line 52: "several machine learning tools are designed". It is good to say "computational tools" because some methods did not use machine learning methods.

5. Line 704: Fig. 3-7B should be Fig. S3-7C

6. Line 250: "the fraction is not as comparable as their dominative fraction in the TME". The "faction" in the two places has different meaning. The author should re-write it. "The immune cells and fibroblasts, on the other hand, are involved in a large number of interactions with a smaller population". This sentence is also confusing.

**Reviewer 2**

**Were you able to assess all statistics in the manuscript, including the appropriateness of statistical tests used?**

Yes, and I have assessed the statistics in my report.

**Comments to author:**

This is a thoughtful and useful comparison of methods.

**Reviewer 3**

**Were you able to assess all statistics in the manuscript, including the appropriateness of statistical tests used?**

Yes, and I have assessed the statistics in my report.

**Comments to author:**

This work proposed a method to detect ligand-receptor pairs and a pipeline to evaluate the cell interactions using spatial transcriptomics. Authors selected datasets that both ST and scRNAseq data are available so that the evaluation can be benefit from both high throughput of scRNAseq and spatial information from ST.

My major concern is on the analytical side

1. A key concept in this work is the long/short range interactions. But I found it hard to understand. Based on the definitions, a short range interaction is when L and R have a smaller w-distance in expression levels -- which means they have similar expression distributions -- and a long distance means they have different distributions. But this should have nothing to do with their spatial distances. I hope author could further explain this. Maybe there is a symbol/presentation issue: L and R should not be 1-D vectors, instead, it should be a 2D distributions w.r.t spatial positions. In that case the spatial permutation makes sense.

2. In the work, authors used CellChatDB tool to analyze the long/short range interactions and prove they can reflect real long/short cell interactions. Here is a logic question, if we can have a good tool to infer real interaction type, then there is no need to define your own long/short range interactions.

3. A way to evaluate it is to directly perform such analysis on ST data and check spatial positions of L-enriched cells and R-enriched cells.

4. Equations on w-distance: the w-distance can be formulated as absolute differences between two cumulative distribution functions. This should be irrelevant to the order of distributions. Why in equation (3) on page 31, you need to calculate it twice?

5. Wasserstein metric: Did you use any normalization or transformation to the expression before calculating the metric?

6. Line 732, I don't understand how ct_distance(cta|ctb)could represent the distance between a and b cell types -- it calculated the average cell distance in a and b separately and take an average of these two distances. There is no interaction term between spot in a and spot in b.

Writing:

1. Maybe I missed this section, in the methods, authors defined L-R interations, cell type- cell type distances, but how to connect these two parts to get results shown in figures?

2. Authors proposed their own way to identify LR pairs, and they also compare the LR results from other approaches. So what's the major focus of this work?

**Reviewer 4**

**Were you able to assess all statistics in the manuscript, including the appropriateness of statistical tests used?**

Yes, and I have assessed the statistics in my report.

**Comments to author:**

The authors compare commonly used single cell RNA sequencing cell-cell interaction inference tools across multiple diverse datasets. I think that this is needed by the research community since much research relies on cell-cell interaction databases and inference tools. I understand there is no ground truth, however, I think that the manuscript would be stronger if the authors generated their own via simulation. Overall, there are a few places where bias is introduced due to a lack of a true ground truth but I think the manuscript is informative and has a lot of useful information.

I recognize this other manuscript is not yet published but it would be useful for the authors to compare and contrast their analysis with Dimitrov et al. (https://doi.org/10.1101/2021.05.21.445160) since there is some overlap and they are both relatively comprehensive evaluations.

CellChatDB was used to assign interaction type. The authors should address how the near/far interaction types defined based on CellChatDB may affect the evaluation metrics. What biases are introduced by this step? It is notable that CellChat had the most stable performance across datasets (line 392).

The ground truth used to calculate F1, precision, and recall is based on interactions identified by 3 or more tools. This can be thought of as more of a measure of consistency than a true ground truth. For instance, if three tools have very similar algorithms and make very similar predictions then they will have much higher evaluation metrics.

I would suggest generating simulated ST data by combining cells to use as an additional ground truth. In this way the authors can design an experiment with known short/long range interactions. Furthermore, I think that this would set this manuscript apart from Dimitrov et al.

Minor comments:

The manuscript seems well written but I did notice a few very minor typos like line 672 "convenient" should be "convenience".

**Authors Response**

**Point-by-point responses to the reviewers’ comments:**

Referee #1

Comments for the Author

Given that increasing attention has been drawn to cell-cell communication analysis in the field of single-cell biology, sysmatical evaluation of computational tools for inferring cell-cell interactions (CCIs) is highly needed. In this manuscript, Liu et al. presented an evaluation framework for systematically benchmarking existing CCI tools. Due to the lack of ground truth, the key assumption of this proposed framework is that spatial distance suggests the interaction tendency of different cell types. The authors proposed a new metric called distance enrichment score (DES) to quantify the coherence between expected and observed spatial distance tendencies by integrating scRNA-seq and spatial transcriptomics (ST) data. After benchmarking 10 cell-cell interaction methods in five matched scRNA-seq and ST datasets, the authors provided suggestions of tools with better performance.

This study provides a timely evaluation framework as well as the guildance for the use of CCI tools, which is valuable to the community of cell-cell communication analysis. However, the current study lacks comparisons with several other tools and also existing tools designed for ST data, which is very important for the guildance of future study. Below is a list of specific comments.

*Reply: We thank the reviewer for appreciating the value of our study. Following the reviewer’s suggestions, we included 6 new CCI tools and 6 additional samples in our evaluation workflow and found the results were in general consistent. Besides, we benchmarked the CCI tools using simulated scRNA-seq, ST datasets, and interactions as ground truth. We also tested the evaluation method provided in CytoTalk and compared it with our method. As suggested by the reviewer, we provided an evaluation pipeline with documentation for adding other new CCI tools. Several descriptions which may cause misunderstanding have been modified and more details on the criteria of parameter choices in different tools were added in the revised manuscript.*

Major:

1. Several other tools designed for scRNA-seq were missing in the benchmarking, such as scConnect (Bioinformatics, 2021), CellCall (NAR, 2021) and Dmonino (bioRxiv 2020.07.24.21853). A baseline method based on the product of ligand and receptor expressions should be also included. In addition, it is of great value to compare the performance against the recently developed tools for ST data, such as CellPhoneDB V3, Giotto (Genome Biology, 2021), Cell2Cell (bioRxiv 2020.11.22.392217) and stLearn (bioRxiv 2020.05.31.125658).

*Reply: Based on the reviewer’s suggestion, we added the following tools in our evaluation workflow, including a baseline method based on the product of averaged ligand and receptor expression between cell types (named LR product), scRNA-seq-based methods including CellCall^[1]^, Domino^[2]^, ST-based methods including CellPhoneDB v3^[3]^, Giotto^[4]^, and stLearn^[5]^. The scConnect returned empty results when we tested it using small-size samples and Cell2Cell^[6]^ does not provide APIs for processing ST data from the 10X Visium platform, so we did not include the two tools in our workflow.*

*Based on the updated DES evaluation result (Figure R1A), several tools, such as CellChat^[7]^, ICELLNET^[8]^, and SingleCellSignalR^[9]^ still have higher ranks similar to the previous result, indicating that the DES metric is stable to evaluate the spatial interaction tendency of predicted interactions. For the updated F1 score ranks with the commonly identified interactions, statistical-based tools, such as CellPhoneDB^[10]^, ICELLNET and iTALK^[11]^ tend to predict more* *common CCIs than network-based or ST-based tools, while CellPhoneDB still has the highest similarity with the commonly identified CCIs (Figure R1B).*

*Interestingly, we found that the ST-based tools that integrate spatial information, such as CellPhoneDB v3, Giotto, and stLearn, did not get significantly higher ranks than the scRNA-seq-based tools for the DES metric. It could be probably explained by the biases introduced by different spatial information integrating methods of these tools. In our evaluation workflow, the spatial distance between each ligand-receptor pair is defined using their expression distribution in the whole ST slides. This kind of distance can reflect the ligand-receptor co-expression from the global slide view and is independent of the potential bias introduced by cell-type annotation. However, in CellPhoneDB v3, Giotto, and stLearn, the spatial distance of each ligand-receptor pair is defined based on the cell type colocalization patterns, which might be significantly affected by wrong cell type mapping or annotations. In our benchmark workflow, the spatial tendency was evaluated using an enrichment score-based method using relative ranks, which will reduce the potential affluence of a few misannotated cells. In addition, those ST-based methods focus more on the ligand-receptor co-expression in a local region rather than the global slide view, which will also cause differences in defining the short and long-range interactions. For the scRNA-seq-based tools, they do not utilize spatial information thus the potential bias in integrating spatial information is the same for all tools. Therefore, although we also benchmarked the performance of ST-based tools, their poor performance might arise from the difference in integrating spatial information, and we recommend benchmarking the scRNA-seq-based tools using our workflow to get more stable results. We have updated the analyses and also discussed the difference between scRNA-seq and ST-based methods in the revised discussion section.*

**Figure R1 Updated DES rank and rank of F-1 score with commonly identified CCIs.**

A. The heatmap shows the rank of each tool’s distance enrichment score (DES) in all 5 datasets and the average DES rank. Tools are sorted by the average DES rank. The actual rank for each tool in each dataset is labeled on the corresponding location. The ‘LR product’ represents the baseline method based on the product of ligand and receptor expressions.

B. The heatmap shows the rank of each tool’s F1 score in all 5 datasets and the average F1 rank. Tools are sorted by the average F1 rank. The actual rank for each tool in each dataset is labeled on the corresponding location. The ‘LR product’ represents the baseline method based on the product of ligand and receptor expressions.

2. In the ST data with predicted cell types, most of the cell types had very few number of cells (less than five in some cases). Since the cell labels were computationally predicted from scRNA-seq data, it is important to evaluate the robustness of the predicted cell labels when performing benchmarking. Do inacurate annotations affect the calculated DES? Since some predicted cell types have extremely small number of cells in several tested datasets, does this affect the calculated DES?

*Reply: The spatial cell-type annotation will influence the distance calculation between cell types and do affect the calculated DES, but it will only have a marginal effect. Two main steps will influence the calculated DES, one is the definition of short/long-range interactions and the other one is the definition of near/far cell-type pairs. The short/long-range interaction definition is based on the expression distribution of ligand and receptors and does not need cell type annotations. In the near/far cell-type definition step, the average distance between cell types will be calculated, and cell-type pairs are clustered into near, median, and far types based on their spatial distance. The clustering information of cell-type pairs will be used for calculated DES, while the absolute distance between cell types and the spot number in a specific cell type does not directly participate in calculating DES. Therefore, a few ambiguous spot annotations will not affect DES too much. But if a large number of spot annotations are inaccurate, that will affect the definition of near/far cell-type pairs and make our evaluation result unstable.*

*It is worth mentioning that although some cell types on ST may have a few spots, the corresponding scRNA-seq could have a richer number of cells for an accurate cell type prediction. In addition, to ensure the robustness of the predicted cell labels, we used STRIDE ^[10]^ for spatial cell-type annotation, which is a topic modeling-based method developed by our group for accurately decomposing and integrating ST slides. In our previous work, we evaluated the performance of STRIDE under different conditions with other published cell-type deconvolution tools, and STRIDE exhibited the overall best performance among all methods and achieved a balance between sensitivity and specificity. Taken together, we conclude that a few misannotated spots will not affect the calculation of DES significantly, and the ST spots annotation based on deconvolution results will ensure the robustness of the cell type annotation.*

3. The authors only selected one patient sample from ST data when integrating each scRNA-seq dataset. Due to the high variability of patient samples, it is necessary to examine the performance using different patient samples for the same biological system. Does the choice of patients affect the consistency?

*Reply: We thank the reviewer for pointing this out, it is necessary to benchmark using more samples to reduce the bias that comes from sample variability. As suggested by the reviewer, we included 6 additional samples in our study, including 1 PDAC sample (GSM4100724), 2 SCC samples (GSM4284321, GSM4284325), 1 human intestine sample (GSM4797918), and 2 human heart samples (6.5PCW_sample8, 6.5PWC_sample10). We compared the evaluation results in each dataset and found that results after adding samples were consistent with the* *previous results (Figure R2A, B, using the human intestine dataset as an example). We also compared the average rank across all datasets before and after adding new samples, CellChat, ICELLNET^[8]^, SingleCellSignalR^[9]^, NicheNet^[11]^, and CellPhoneDB^[12]^ had the top 5 rank, which is also consistent with our previous conclusions (Figure R2C, D). In summary, the DES metric for evaluating the CCI tools does not change significantly after adding more samples. We have updated the figures in the revised manuscript.*

**Figure R2 DES metric gives consistent results after adding new samples.**

A. Distance Enrichment Score (DES) rank in the human intestinal dataset of sample ST_A4_GSM4797919 before adding tools and samples.

B. DES rank in the human intestinal dataset of all samples (ST_A3_GSM4797918, ST_A4_GSM4797919) after adding tools and samples. The box plot reflects the overall distribution of DES across all samples. Tools are sorted by the median DES. The ‘LR product’ represents the baseline method based on the product of ligand and receptor expressions.

C. The heatmap shows the rank of each tool’s DES in all 5 datasets before adding tools and samples. Tools are sorted by the average DES rank. The actual rank for each tool in each dataset is labeled on the corresponding location.

D. The heatmap shows the rank of each tool’s DES in all 5 datasets after adding tools and samples. Tools are sorted by the average DES rank. The actual rank for each tool in each dataset is labeled on the corresponding location. The ‘LR product’ represents the baseline method based on the product of ligand and receptor expressions.

4. CytoTalk (Science Advances, 2021) provided a similar way to benchmark CCI tools by integrating scRNA-seq and ST data. It is valuable to include the evaluation method used in this paper.

*Reply: We thank the reviewer for this helpful suggestion. We followed the description in CytoTalk to evaluate these tools based on mutual information between the scRNA-seq and ST datasets and tested the method on the human intestine dataset. The CytoTalk evaluation method categorized spot pairs into close and distal groups according to the spatial distance. Then it computed the mutual information for predicted LR pairs in these two groups and compared the differences between the two distributions of mutual information using the one-sided Kolmogorov-Smirnov test. The result of the CytoTalk evaluation method gave out a similar result to our DES evaluation method (Figure R3A, B). NicheNet, CellChat, and SingleCellSignalR were consistently ranked in the top 5 no matter evaluated using the CytoTalk metric or our DES metric. CellPhoneDB, CellPhoneDBv3, Giotto, and CellCall showed consistently bottom ranks in both metrics (Figure R3A, B). Considering the ranks of the two metrics were in general similar, we included the evaluation of the CytoTalk metric in the revised supplementary figures.*

**Figure R3 Evaluation results of the CytoTalk and DES metrics are similar.**

A. Evaluation rank in human intestinal dataset using CytoTalk evaluation method. The box reflects the overall statistical significance across all samples and cell types. Tools are sorted by the median statistical significance. The ‘LR product’ represents the baseline method based on the product of ligand and receptor expressions.

B. DES rank in the human intestinal dataset. The box plot reflects the overall distribution of DES across all samples. Tools are sorted by the median DES. The ‘LR product’ represents the baseline method based on the product of ligand and receptor expressions.

5. While the authors provided a well-documented evaluation framework, it is worth providing a complete example pipeline for facilitating the comparison analysis when new tools are developed in the future. The authors should (1) provide a documented guildance when new tools are adding into the comparison analysis; and (2) provide the benchmarking codes for all tested datasets when the scRNA-seq data and cell labels of ST data are given.

*Reply: We have packaged the benchmark workflow with detailed documentation at https://github.com/wanglabtongji/CCI. All the simulated and real data benchmarking results are included in the workflow. We provided the installation codes as well as demo running scripts for all the CCI tools included in our study, and also packaged the human intestine dataset as an example dataset in the workflow. Users can download other real datasets included in our study through the corresponding links and generate the simulated datasets through the scripts that we provided. In addition, we provided documented guidance for adding new tools. Users could* *generate the predicted CCIs using new tools and provide the result to our workflow, and our workflow will output the DES metrics based on the given scRNA-seq and ST data. The repository links have been included in the revised manuscript.*

Minor:

1. Is there any reason why NicheNet performs well in two tumor datasets but not in the other two datasets?

*Reply: One probable explanation is that the prior model used in NicheNet has a bias toward gene regulations and cell-cell interactions in the tumor microenvironment. NicheNet uses a network model with prior information on potential ligand-target links to predict activate interactions between cell types. The regulation potential between ligands and target genes in this model was calculated based on an integrated network that aggregates public data resources on ligand-receptor, signaling pathways, and gene regulatory relationships, which could be biased if there are more tumor-associated interactions in the public data resources.*

2. Is there any reason why the authors used the top 50 ligands in NicheNet but the top 100 in ICELLNET? In scMLnet, the authors metioned "To control the predicted interaction number". Can the authors add more details on the criteria of the choice of parameters for selecting inferred interactions in different methods.

*Reply: Normally, we will use the recommended values for each method as the choice of parameters. If no default or recommended values are supplied, we will try to adjust several parameters to make these tools have similar numbers of predicted interaction numbers with other tools. NicheNet needs to first predict active ligands between the two cell types and then select active target genes and receptors based on those ligands. And the top 50 ligands limit is set to the maximum number of active ligands allowed by the algorithm. The correct threshold used in ICELLNET is top 200, we have corrected it in the revised manuscript. In ICELLNET, for a specific sender cell type, it will return all possible interactions between all other cell types in one matrix and rank them by interaction score. The top 200 here is set as the limit of the maximum number of selected interactions in multiple cell type pairs, so we need to set this parameter larger than NicheNet to make sure that each cell type pair have enough interactions. In summary, the aim of different parameter settings is to generate comparable numbers of significant interactions between different tools. We have provided more detailed explanations in the revised method section.*

3. In line 705, the authors used the top 10% to define the long and short range of interactions, but used the top 15% in line 844. Why not use the same parameter?

*Reply: The PDAC dataset has a relatively lower number of detected interactions than other datasets due to its lower gene capture rate. There is no adequate number of long and short-range interactions in the PDAC dataset if using the top 10% as the cutoff. To get enough common interactions for benchmarking, we increased the cutoff to the top 15%. But for other datasets, we still used the top 10% as the cutoff to define long and short-range interactions. We have added the explanation in the revised method section.*

4. Line 52: "several machine learning tools are designed". It is good to say "computational tools" because some methods did not use machine learning methods.

*Reply: We have modified the description to “computational tools” in the revised manuscript.*

5. Line 704: Fig. 3-7B should be Fig. S3-7C

*Reply: We corrected the figure citation in the revised manuscript.*

6. Line 250: "the fraction is not as comparable as their dominative fraction in the TME". The "fraction" in the two places has different meaning. The author should re-write it. "The immune cells and fibroblasts, on the other hand, are involved in a large number of interactions with a smaller population". This sentence is also confusing.

*Reply: We have re-written these sentences in the revised manuscript as follows.*

- *"the fraction is not as comparable as their dominative fraction in the TME.” changed to “Interestingly, although malignant cells are abundant in the TME according to the ST data, the number of interactions of malignant cells predicted by different tools did not display a comparable dominate fraction.”*
- *"The immune cells and fibroblasts, on the other hand, are involved in a large number of interactions with a smaller population.” changed to “The less abundant immune cells and fibroblasts, on the other hand, are involved in a large number of interactions.”*

Referee #2

Comments for the Author

This is a thoughtful and useful comparison of methods.

*Reply: We thank the reviewer for their high recognition of our study.*

Referee #3

Comments for the Author

This work proposed a method to detect ligand-receptor pairs and a pipeline to evaluate the cell interactions using spatial transcriptomics. Authors selected datasets that both ST and scRNAseq data are available so that the evaluation can be benefit from both high throughput of scRNAseq and spatial information from ST.

*Reply: We appreciate the reviewer for seeing the value of our study and their helpful suggestions. Based on this and other reviewers’ comments, we added more details for the Wasserstein distance calculation, short/long-range interaction definition, and updated the formula for computing cell type distances in the revised method section. Besides, we also added comparisons of new CCI tools and samples, benchmarked the performance using simulated datasets as ground truth, and packed the workflow for future evaluation of new CCI tools. The detailed changes were as follows.*

Major:

1. A key concept in this work is the long/short range interactions. But I found it hard to understand. Based on the definitions, a short-range interaction is when L and R have a smaller w-distance in expression levels -- which means they have similar expression distributions -- and a long distance means they have different distributions. But this should have nothing to do with their spatial distances. I hope author could further explain this. Maybe there is a symbol/presentation issue: L and R should not be 1-D vectors, instead, it should be a 2D distributions w.r.t spatial positions. In that case the spatial permutation makes sense.

*Reply: Thank you for your suggestion! L and R are indeed 2D distributions that represent gene expression levels in corresponding spatial coordinates. When we calculate the w-distance, we will first generate a matrix D to record the Euclidean distance between each data point in L and R according to their spatial coordinates. In this case, we can flatten L and R into 1D vectors while still keeping their spatial distances in matrix D. We have modified our descriptions in the revised manuscript to avoid misunderstanding.*

2. In the work, authors used CellChatDB tool to analyze the long/short range interactions and prove they can reflect real long/short cell interactions. Here is a logic question, if we can have a good tool to infer real interaction type, then there is no need to define your own long/short range interactions.

*Reply: The long/short range interactions in the CellChatDB are not predicted by CellChat, but rather are L-R interaction information collected from the public literature or databases. It is possible that the literature collected long/short-range interactions do not have tissue specificities. Therefore, we still need to define our own long/short-range interactions based on the real scRNA-seq and ST datasets from the corresponding tissues. And the long/short* *interactions from CellChatDB could be used as an additional reference to validate the accuracy of our definitions. In Figure 1d, we could see that although the short-range interactions are mostly enriched in cell-cell contact, and the long-range interactions are mainly secreted signaling, however, there are also a few exceptions indicating potential tissue specificities. We have added the descriptions in the revised method section.*

3. A way to evaluate it is to directly perform such analysis on ST data and check spatial positions of L-enriched cells and R-enriched cells.

*Reply: We thank the reviewer for this suggestion. We agree that it’s a direct way to evaluate the CCI tools using ST data, however, we do not apply the strategy for the following reasons. First, the current ST datasets we used are mostly from the 10X Visium platform with a resolution of 55um-100um, which is not at the single-cell resolution. Each spot from the ST slides could be regarded as a mixture of 6-10 cells on average. While most CCI tools were designed for single-cell analyses, the gene expression distribution in the mixed spots should be different from single-cells thus is not appropriate to directly apply many statistical-test-based CCI methods. Second, although there are computational methods that could map the single-cells to the ST data and predict their spatial locations, however, directly comparing the spatial positions of L-enriched cells and R-enriched cells are highly dependent on the quality of predicted spatial locations of single-cells, which could be biased for cells with low coverage. Our methods first define the long/short-range interactions based on ST data, then define the distance between different cell types by integrating the scRNA-seq and ST data with STRIDE, a method that we previously developed for mapping scRNA-seq cells with ST spots. By leveraging the information at the cell type level, and also comparing the consistency using an enrichment-based method, our methods are robust to potential wrong spatial mapping results for low coverage cells. Finally, in the revised manuscript, we have also compared the performance of several ST-based methods using our DES metrics (Figure R1, Point 1 to #Reivewer 1), however, the performance of the ST-based tools is not always better than scRNA-seq-based tools even with spatial information incorporated. We discussed the difference that might arise from different ways of incorporating spatial information and the way of defining long/short-range interactions at the local or global level. Taken together, we do not directly apply CCI methods directly on ST datasets due to inappropriate data distributions and potential errors in single-cell mapping. We have updated these analyses in the revised discussion section.*

4. Equations on w-distance: the w-distance can be formulated as absolute differences between two cumulative distribution functions. This should be irrelevant to the order of distributions. Why in equation (3) on page 31, you need to calculate it twice?

*Reply: Since it is time-consuming to compute the Wasserstein distance in two-dimensional space, the frequently used solution is to compute the Wasserstein distance using the Sinkhorn algorithm. The Sinkhorn algorithm is a regularized version of Wasserstein distance and it computes much faster by estimating the optimal solution through iteration. Due to the addition of entropic regularization terms and iterations, the results of the Sinkhorn algorithm on different orders of distributions will be slightly different (< 1 most of the time). In this case, we decided to compute the Wasserstein distance in both the L-R and R-L directions and used their average as the final value. We thank the reviewer for pointing this out, and we have updated the descriptions in the revised method section.*

5. Wasserstein metric: Did you use any normalization or transformation to the expression before calculating the metric?

*Reply: We used sctransform^[15]^ to normalize the count matrix before calculating the Wasserstein metric.*

6. Line 732, I don't understand how ct_distance(cta|ctb) could represent the distance between a and b cell types -- it calculated the average cell distance in a and b separately and take an average of these two distances. There is no interaction term between spot in a and spot in b.

*Reply: We are sorry for our original symbols caused a potential misunderstanding. We have modified the formula in the revised manuscript as follows.*

$$ct\_distance\left( ct_{a} | ct_{b} \right) = \left( \frac{\sum_{i=1}^{n_{a}} \min_{j=1,2..,n_{b}} dis(spot_{ai}, spot_{bj})}{n_{a}}+\frac{\sum_{j=1}^{n_{b}} \min_{i=1,2,\ldots,n_{a}} dis(spot_{bj}, spot_{ai})}{n_{b}} \right)/2$$

*where* $ct_{a}$ *and* $ct_{b}$ *stand for the cell type a and cell type b;* $n_{a}$*,* $n_{b}$ *are the spot number of corresponding cell type;* $spot_{ai}$ *is the ith spot in cell type a; function* $dis$*() computes the Euclidean distance between two spots. To make the calculation of the cell type distance clear, we have added a schematic illustration (Figure R4). All these contents have been updated in the revised manuscript.*

**Figure R4 Schematic illustration of computing cell type distance.**

The Schematic figure illustrates the procedure for computing cell type distance. Each point indicates a spot in the ST data, the color represents the cell type. The black dotted line shows the Euclidean distance between connected spots, while the red line indicates that the connected spots have the minimum distance than other black dotted line connected spots. The distance between cell types can be quantified through the mean of minimum Euclidean distances between spots.

Minor:

1. Maybe I missed this section, in the methods, authors defined L-R interations, cell type- cell type distances, but how to connect these two parts to get results shown in figures?

*Reply: We connected short/long-range interactions and near/far cell-type pairs using a method similar to gene set enrichment analyses (GSEA) in the “Distance Enrichment Score” section. In short, the defined short/long-range L-R interactions could be treated as expected interactions, and the significant interactions identified from near/far cell types could be treated as observed interactions and ranked by P-values or other significant scores from CCI tools. The DES scores of near cell types were calculated against short-range interactions and the far cell types were calculated against long-range interactions using the enrichment score method similar to the GSEA definition. The overall DES scores for different cell type pairs were finally averaged to generate the final metric.*

2. Authors proposed their own way to identify LR pairs, and they also compare the LR results from other approaches. So what's the major focus of this work?

*Reply: The major focus of our work is to evaluate the accuracy of scRNA-seq-based CCI tools by integrating spatial information from ST data. The basic hypothesis of this work is that the near cell types should have more short-range interactions, and the far cell types should have more long-range interactions. The short and long-range LR pairs defined by our methods are* *generated from ST data and served as evaluation references. They reflect the known/expected spatial distance tendencies of LR pairs in the real tissues. The LR results from CCI tools were predicted without spatial information, and comparing the predicted LR results from CCI tools with our LR pairs could evaluate whether the predicted interactions were consistent with their spatial locations, as LR interactions that are consistent with their spatial locations are more likely to occur in real tissues. The benchmark strategy here provides a simple and experiment-free way for validating potential interactions between two cell types. In summary, our study not only provides a way for evaluating the accuracy of CCI prediction based on spatial information but also could guide the future development of more accurate CCI predicting tools by directly including spatial information.*

Referee #4

Comments for the Author

The authors compare commonly used single cell RNA sequencing cell-cell interaction inference tools across multiple diverse datasets. I think that this is needed by the research community since much research relies on cell-cell interaction databases and inference tools. I understand there is no ground truth, however, I think that the manuscript would be stronger if the authors generated their own via simulation. Overall, there are a few places where bias is introduced due to a lack of a true ground truth but I think the manuscript is informative and has a lot of useful information.

*Reply: We thank the reviewer for seeing the value of our work to the research community. Based on the reviewer’s suggestions, we generated simulated datasets for benchmarking and added more CCI tools and samples for a comprehensive evaluation. We also compared our work with another relative evaluation work, although the focuses of these two works are different, there are still several consistent findings. We also updated the descriptions of the metrics of commonly identified interactions, and added more details on short/long-range interaction definition, DES score calculation, and parameter selection for different CCI tools. Finally, we packed our workflow in Github and provided documents for adding new CCI tools for future comparison.*

Major:

1. I recognize this other manuscript is not yet published but it would be useful for the authors to compare and contrast their analysis with Dimitrov et al. (https://doi.org/10.1101/2021.05.21.445160) since there is some overlap and they are both relatively comprehensive evaluations.

*Reply: The evaluation work from Dimitrov et al.^[14]^ mainly focused on comparing the L-R databases used by different CCI tools and analyzed the usage of different databases in influencing CCI prediction. Their results suggested that both the CCI tools and database can significantly impact the predicted CCIs. The coverage of interactions in different databases is biased toward specific subcellular locations and functional categories which will introduce different biases in CCI predicting. These analyses are complementary to our benchmarking study, which mainly focused on evaluating the accuracy of different CCI prediction tools based on the integration of ST data. Also, in our study, we only used the CCI database from CellChatDB to control the impact of different L-R databases.*

*Both Dimitrov et al. and our study evaluated the performance of CCI tools by comparing the results with common interactions. We have a similar conclusion that the shared interactions between different CCI tools were relatively low, even after we run different tools using the same L-R database. In our benchmark, we also found that the statistical-based methods and network-based methods show different overlaps with common interactions, with statistical-based methods tend to show more consistent interactions between different methods. Besides, we also used the simulated datasets as well as integrated the spatial information from ST datasets to provide a more comprehensive benchmark for the accuracy of predicted CCIs. In summary, our study has some overlap with Dimitrov et al., but we provided more strategies and comprehensive evaluations on the accuracy of CCI predictions, while Dimitrov et al are more focused on benchmarking the usage of different CCI databases. We have included these analyses in the revised discussion section.*

2. CellChatDB was used to assign interaction type. The authors should address how the near/far interaction types defined based on CellChatDB may affect the evaluation metrics. What biases are introduced by this step? It is notable that CellChat had the most stable performance across datasets (line 392).

*Reply: We defined short/long-range interaction types by calculating the Wasserstein distance between ligand and receptor distributions based on the ST data. And we did not directly use the interaction types in CellChatDB to assign short/long-range interactions. The interaction types in CellChatDB are only used as evidence to support the rationality of our short/long-range interaction types, thus will not introduce biases in the evaluation workflow. In addition, although the L-R database used in our database was mainly from CellChatDB, we also included the L-R interactions from other CCI databases to make the database we used as comprehensive as possible. However, for some tools with complex database structures such as NicheNet, scMLnet, Connectome, and CellCall, it is hard for us to replace their databases with our curated database and we still use the database from the original methods, which will introduce some bias. For most other tools, the potential biases from the database can be controlled. We have added the descriptions in the revised method section.*

3. The ground truth used to calculate F1, precision, and recall is based on interactions identified by 3 or more tools. This can be thought of as more of a measure of consistency than a true ground truth. For instance, if three tools have very similar algorithms and make very similar predictions then they will have much higher evaluation metrics.

*Reply: We understand the concern of the reviewer. In the revised manuscript, we did not use the metric of commonly identified interactions as measures of ground truth in our evaluation workflow. These metrics were only used to reflect the similarity of results from different tools. Instead, we generated 15 simulated scRNA-seq and ST datasets, and evaluate the accuracy of predicted CCIs by a semi-synthetic strategy using the simulated datasets (see Point 4 for this reviewer). And the final accuracy rank combines the DES score from the simulated datasets, as well as the DES score from the real datasets. We have updated the revised manuscript and figures accordingly.*

4. I would suggest generating simulated ST data by combining cells to use as an additional ground truth. In this way the authors can design an experiment with known short/long range interactions. Furthermore, I think that this would set this manuscript apart from Dimitrov et al.

*Reply: We thank the reviewer for the helpful suggestion. We simulated 15 paired scRNA-seq and ST data with known overexpressed ligand-receptor pairs as suggested by the reviewer. The simulation workflow is as follows (Figure R5A):*

***Step 1: Simulation of scRNA-seq and ST data.***

*In each simulation round, 4 cell types in ST data are selected and are assigned with the near/far cell-type pair defined in the original ST data (Figure R5B). For each cell type, we randomly selected cells from the same cell type in the original scRNA-seq data, and the number of selected cells is based on the number of spots to ensure each spot has approximately 2~5 cells after mapping scRNA-seq cells to ST. Next, the randomly selected cells will be randomly mapped to each spot according to their cell types and replace the original expression of the spots. To keep the real spatial cell-type structure in ST data, we did not change the original coordinates of selected spots.*

***Step 2: Simulation of interacted L-R pairs by semi-synthetic strategy***

*Then for each near/far cell-type pair, 30 interactions are randomly selected, and a semi-synthetic method ^[15]^ is applied to scRNA-seq data to replace original expression signals of selected ligand and receptor genes with overexpressed signals.*

***Step 3: Filtering simulated interactions to keep short/long-range interaction consistent with the spatial distance of cell type pairs***

*Having simulated ST data, short/long-range interactions can be defined following the same procedure used in the real data. Finally, both simulated scRNA-seq and ST data will be re-modified, only short-range interactions with overexpressed signals will be kept in the near cell-type pairs, and the same for long-range interactions in the far cell-type pairs. After filtering, around 10 simulated interactions will be kept in each cell-type pair (Figure R5C). For the kept simulated interactions, if available, corresponding transcription factors and target genes will also be selected and overexpressed so that the tools based on the transcription factors and targets could be also used.*

*For the 3 different biological systems used in our study (tumor microenvironment, mouse brain, and human organs), we selected one sample of data for each biology system and performed simulation 5 rounds for each sample. In the end, we evaluated these tools using 15 simulated datasets (Figure R5D, E). The evaluation results on the simulated datasets (Figure R5D) were consistent with results on the real datasets (Figure R1) on the top-ranked tools. We also evaluated these tools by simply comparing the overlaps between predicted interactions with simulated interactions. Using simulated interactions in each cell-type pair as the positive set, we computed the F1 score for each tool. For top-ranked tools, the results of F1 rank were still consistent with the results of DES rank (Figure R5E). In summary, these results suggest that our DES metric, as well as the simulation strategy, are robust in evaluating the performance of CCI prediction tools.*

**Figure R5 Evaluation using simulated datasets.**

A. The Schematic illustration of data simulation procedure. The long box indicates the LR pair between two cell types, where its face color represents interaction type and edge color represents expression state. The rounded rectangles indicate cell types and edge color represents distance type between cell types.

B. The example of randomly selected cell types in ST data. Each point represents a spot, and different colors represent different cell types.

C. The bar plot displays the number of available simulated short/long-range interactions in selected near/far cell-type pairs after re-modified. The kept short-range interactions are colored in blue; kept long-range interactions are colored in orange; unavailable interactions are colored in gray.

D. The DES ranks of selected tools evaluating using 15 simulated datasets. Tools are sorted by the median DES.

E. The F1 ranks of selected tools evaluating using 15 simulated datasets using simulated interactions as positive set. Tools are sorted by the median F1 score.

Minor:

1. The manuscript seems well written but I did notice a few very minor typos like line 672 "convenient" should be "convenience".

*Reply: We have corrected these typos in the revised manuscript.*

***Reference***

1. *Zhang Y, Liu T, Hu X, Wang M, Wang J, Zou B, et al.. CellCall: integrating paired ligand-receptor and transcription factor activities for cell-cell communication. Nucleic Acids Research. Nucleic Acids Research; 2021;49:8520–34.*
2. *Cherry C, Maestas DR, Han J, Andorko JI, Cahan P, Fertig EJ, et al.. Intercellular signaling dynamics from a single cell atlas of the biomaterials response. 2020..*
3. *Garcia-Alonso L, Handfield L-F, Roberts K, Nikolakopoulou K, Fernando RC, Gardner L, et al.. Mapping the temporal and spatial dynamics of the human endometrium in vivo and in vitro. Nature Genetics. Nature Genetics; 2021;53:1698–711.*
4. *Dries R, Zhu Q, Dong R, Eng C-HL, Li H, Liu K, et al.. Giotto: a toolbox for integrative analysis and visualization of spatial expression data. Genome Biology. Genome Biology; 2021;22.*
5. *Pham D, Tan X, Xu J, Grice LF, Lam PY, Raghubar A, et al.. stLearn: integrating spatial location, tissue morphology and gene expression to find cell types, cell-cell interactions and spatial trajectories within undissociated tissues. 2020..*
6. *Armingol E, Ghaddar A, Joshi CJ, Baghdassarian H, Shamie I, Chan J, et al.. Inferring a spatial code of cell-cell interactions across a whole animal body. 2020..*
7. *Jin S, Guerrero-Juarez CF, Zhang L, Chang I, Ramos R, Kuan C-H, et al.. Inference and analysis of cell-cell communication using CellChat. Nature Communications. Nature Communications; 2021;12.*
8. *Noël F, Massenet-Regad L, Carmi-Levy I, Cappuccio A, Grandclaudon M, Trichot C, et al. Dissection of intercellular communication using the transcriptome-based framework ICELLNET. Nature Communications. 2021;12.*
9. *Cabello-Aguilar S, Alame M, Kon-Sun-Tack F, Fau C, Lacroix M, Colinge J. SingleCellSignalR: inference of intercellular networks from single-cell transcriptomics. Nucleic Acids Research. 2020;48:e55.*
10. *Efremova M, Vento-Tormo M, Teichmann SA, Vento-Tormo R. CellPhoneDB: inferring cell–cell communication from combined expression of multi-subunit ligand–receptor complexes. Nature Protocols. 2020;15:1484–506.*
11. *Wang Y, Wang R, Zhang S, Song S, Jiang C, Han G, et al. iTALK: an R Package to Characterize and Illustrate Intercellular Communication. bioRxiv. 2019; 507871. doi: 10.1101/507871.*
12. *Sun D, Liu Z, Li T, Wu Q, Wang C. STRIDE: accurately decomposing and integrating spatial transcriptomics using single-cell RNA sequencing. Nucleic Acids Research; 2022;50:e42.*
13. *Browaeys R, Saelens W, Saeys Y. NicheNet: modeling intercellular communication by linking ligands to target genes. Nature Methods. 2020;17:159–62.*
14. *Efremova M, Vento-Tormo M, Teichmann SA, Vento-Tormo R. CellPhoneDB: inferring cell-cell communication from combined expression of multi-subunit ligand-receptor complexes. Nature Protocols. 2020;15:1484–506.*
15. *Hafemeister C, Satija R. Normalization and variance stabilization of single-cell RNA-seq data using regularized negative binomial regression. Genome Biology. Genome Biology; 2019;20.*
16. *Dimitrov D, Türei D, Boys C, Nagai JS, Ramirez Flores RO, Kim H, et al.. Comparison of Resources and Methods to infer Cell-Cell Communication from Single-cell RNA Data. 2021..*
17. *Wieder C, Lai RP, Ebbels T. Single sample pathway analysis in metabolomics: performance evaluation and application. 2022.*

**Second round of review**

**Reviewer 1**

The authors have addressed most of my concerns, but I still have the following concerns. I hope the authors can well address them.

The authors compared different methods using the commonly identified interactions. However, the results were likely biased dependent on the selected methods. Similar methods very likely shared the more common interactions. Thus, it may not be a fair metric to evaluate different methods. While I noticed that the authors mentioned this limitation in the Discussion in this revision, it was better to mention this point in the Results section as well.

I do not think the authors have answered my concern: "Since some predicted cell types have extremely small number of cells in several tested datasets, does this affect the calculated DES?". The inference of cell-cell communication was based on the inferred cell types/clusters. But if some predicted cell clusters have extremely small number of cells/spots, it may induce false interactions. For example, high expression of ligands or receptors in these clusters likely lead to stronger interactions compared to other paired cell types. But these may be false interactions due to the inaccurate prediction of cell labels or noisy data. If these cell clusters were removed from the analysis, will the DES change?

**Reviewer 3**

Authors addressed my questions. I appreciate their efforts.

**Reviewer 4**

The manuscript has been significantly improved and would make an informative addition to CCI literature.

**Authors Response**

**Point-by-point responses to the reviewers’ comments:**

Referee #1

Major:

1. The authors compared different methods using the commonly identified interactions. However, the results were likely biased dependent on the selected methods. Similar methods very likely shared the more common interactions. Thus, it may not be a fair metric to evaluate different methods. While I noticed that the authors mentioned this limitation in the Discussion in this revision, it was better to mention this point in the Results section as well.

*Reply: We thank the reviewer for this helpful suggestion. We added more discussions of the potential bias of the commonly identified interaction metric in the Results section along with our previous discussions in the “Commonly identified interaction metric ranks” subsection.*

2. I do not think the authors have answered my concern: "Since some predicted cell types have extremely small number of cells in several tested datasets, does this affect the calculated DES?". The inference of cell-cell communication was based on the inferred cell types/clusters. But if some predicted cell clusters have extremely small number of cells/spots, it may induce false interactions. For example, high expression of ligands or receptors in these clusters likely lead to stronger interactions compared to other paired cell types. But these may be false interactions due to the inaccurate prediction of cell labels or noisy data. If these cell clusters were removed from the analysis, will the DES change?

*Reply: We agree with the reviewer that the predicted cell clusters with an extremely small number of spots might induce false results. Actually, to control the potential risk, we have already removed the rare cell types (the cell type with the number of spots less than 5) while calculating the DES in the previous submission. To better address the influence of rare cell types on the calculated DES, we re-calculated the DES with the rare cell types added back and compared the results with the original ones in the human heart dataset and human intestine dataset (Figure R1). We added the immune cells back while calculating DES in the human heart dataset, and the ranks of CCI tools did not have significant changes (Figure R1A, B, C). In the human intestine dataset, after adding the pericytes back, the DES ranks were slightly changed but the stLearn, SingleCellSignalR, and CellChat were still top-ranked (Figure R1D, E, F). In summary, these analyses suggest that our DES ranks are robust. The effect of rare cell types is marginal and we have controlled the risk by removing these rare cell types while calculating DES.*
